# Spatial transcriptomics analysis identifies a tumor-promoting function of the meningeal stroma in melanoma leptomeningeal disease

**DOI:** 10.1016/j.xcrm.2024.101606

**Published:** 2024-06-11

**Authors:** Hasan Alhaddad, Oscar E. Ospina, Mariam Lotfy Khaled, Yuan Ren, Ethan Vallebuona, Mohammad Baraa Boozo, Peter A. Forsyth, Yolanda Pina, Robert Macaulay, Vincent Law, Kenneth Y. Tsai, W. Douglas Cress, Brooke Fridley, Inna Smalley

**Affiliations:** 1 Department of Metabolism and Physiology, Moffitt Cancer Center, Tampa, FL, USA; 2 Department of Biostatistics and Bioinformatics at the Moffitt Cancer Center, Tampa, FL, USA; 3 Department of Biochemistry, Faculty of Pharmacy, Cairo University, Cairo, Egypt; 4 Cancer Biology Ph.D. Program, University of South Florida, Tampa, FL, USA; 5 Department of Tumor Biology, Moffitt Cancer Center, Tampa, FL, USA; 6 Department of NeuroOncology, Moffitt Cancer Center, Tampa, FL, USA; 7 Department of Pathology, Moffitt Cancer Center, Tampa, FL, USA; 8 Department of Molecular Oncology at the Moffitt Cancer Center, Tampa, FL, USA; 9 Division of Health Services & Outcomes Research, Children’s Mercy Hospital, Kansas City, MO 64108, USA; 10 Department of Cutaneous Oncology at the Moffitt Cancer Center, Tampa, FL, USA

**Keywords:** brain, leptomeninges, CSF, melanoma, leptomeningeal disease, metastasis, drug resistance, MAPK therapy, central nervous system metastasis, pia

## Abstract

Leptomeningeal disease (LMD) remains a rapidly lethal complication for late-stage melanoma patients. Here, we characterize the tumor microenvironment of LMD and patient-matched extra-cranial metastases using spatial transcriptomics in a small number of clinical specimens (nine tissues from two patients) with extensive *in vitro* and *in vivo* validation. The spatial landscape of melanoma LMD is characterized by a lack of immune infiltration and instead exhibits a higher level of stromal involvement. The tumor-stroma interactions at the leptomeninges activate tumor-promoting signaling, mediated through upregulation of SERPINA3. The meningeal stroma is required for melanoma cells to survive in the cerebrospinal fluid (CSF) and promotes MAPK inhibitor resistance. Knocking down SERPINA3 or inhibiting the downstream IGR1R/PI3K/AKT axis results in tumor cell death and re-sensitization to MAPK-targeting therapy. Our data provide a spatial atlas of melanoma LMD, identify the tumor-promoting role of meningeal stroma, and demonstrate a mechanism for overcoming microenvironment-mediated drug resistance in LMD.

## Highlights

• Melanoma LMD has little immune infiltration but higher stromal involvement
• Meningeal stroma is required for melanoma cells to survive in the CSF environment
• Tumor-promoting effects of meningeal stroma are mediated via upregulation of SERPINA3
• Disrupting SERPINA3 signaling sensitizes LMD to MAPK inhibition

## Graphical abstract

**Figure:**
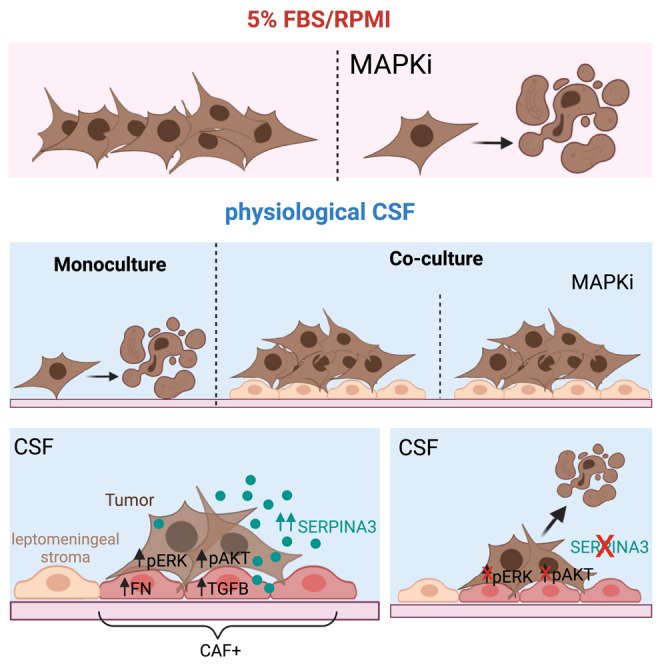

## Teaser

Alhaddad, Ospina, Khaled et al. profile melanoma leptomeningeal disease using spatial transcriptomics. This study identifies a tumor-promoting role of meningeal stroma, mediated through SERPINA3. The tumor-stroma interactions support melanoma survival in CSF and promote resistance to MAPK therapy. These findings identify a targetable vulnerability in leptomeningeal disease.

## Introduction

Leptomeningeal metastasis is a devastating terminal complication occurring in 5%–8% of melanoma patients and remains a major clinical challenge to the treatment of late-stage melanoma patients.^1^^,^^2^^,^^3^ Even with aggressive treatment, the overall survival for melanoma patients with leptomeningeal disease (LMD) has not altered in several decades and is typically measured in weeks to a few months.^1^^,^^3^ Despite significant advancements in the treatment of metastatic melanoma using MAPK-targeting therapies and checkpoint inhibitors at extra-cranial sites or even within the brain parenchyma, the majority of tumors at the leptomeninges do not respond to therapy.^3^^,^^4^ Due to the diffuse nature of the disease, inaccessible site of the tumor, rapid tumor progression, and technological limitations, little is known about the biology of melanoma LMD. We have previously demonstrated that cerebrospinal fluid (CSF) from patients with LMD can modulate BRAF inhibitor responses and may contribute to drug resistance.^5^ Single-cell RNA sequencing characterization of the CSF microenvironment has shown an immune-suppressed cellular landscape in LMD compared with other sites of disease.^6^ Therefore, the leptomeninges likely serve as a "sanctuary" site for melanoma cells treated with targeted kinase inhibitors and checkpoint inhibitor therapies. However, these studies only show us a glimpse into the fluid microenvironment of LMD and do not provide a characterization of the leptomeningeal tissues. No previous studies have examined the spatial microenvironment of LMD, and none have compared melanoma tumors adherent to the leptomeninges to extra-cranial sites.

In melanoma LMD, the tumor cells seed to the CSF space and the membrane coverings of the brain and spinal cord termed the leptomeninges.^1^^,^^2^ The leptomeninges consist of the pia mater, the arachnoid mater, and the CSF space. The pia mater covers the brain’s surface and consists of ICAM1- and SLC38A2-expressing fibroblast-like meningeal cells held together by tight junctions on top of a basement membrane.^7^^,^^8^ Like melanocytes, these specialized meningeal cells arise from mesenchymal tissues in the neural tube.^8^ Many studies have examined the roles of the meningeal cells in neural development and inflammation, but their role in cancer has never been explored.^7^^,^^9^^,^^10^^,^^11^ The CSF environment is metabolically harsh compared with other sites of disease. Healthy CSF generally lacks micronutrients or proteins,^12^ leading us to question how the rapidly growing tumor cells could survive in such an environment. We wondered if the specialized fibroblast-like cells forming the meninges could play a pro-tumorigenic role similar to cancer-associated fibroblasts (CAFs) in the LMD microenvironment. In this manuscript, we perform spatial transcriptomic profiling comparing leptomeningeal tissues with patient-matched extra-cranial melanoma metastases. We show that CAF-like activation of the LMD stroma promotes the survival of the tumor cells in the CSF environment and renders them insensitive to MAPK-targeting therapies. These data identify a mechanism for overcoming the stroma-induced MAPK inhibitor resistance.

## Results

### The spatial landscape of melanoma LMD

**Figure 1:**
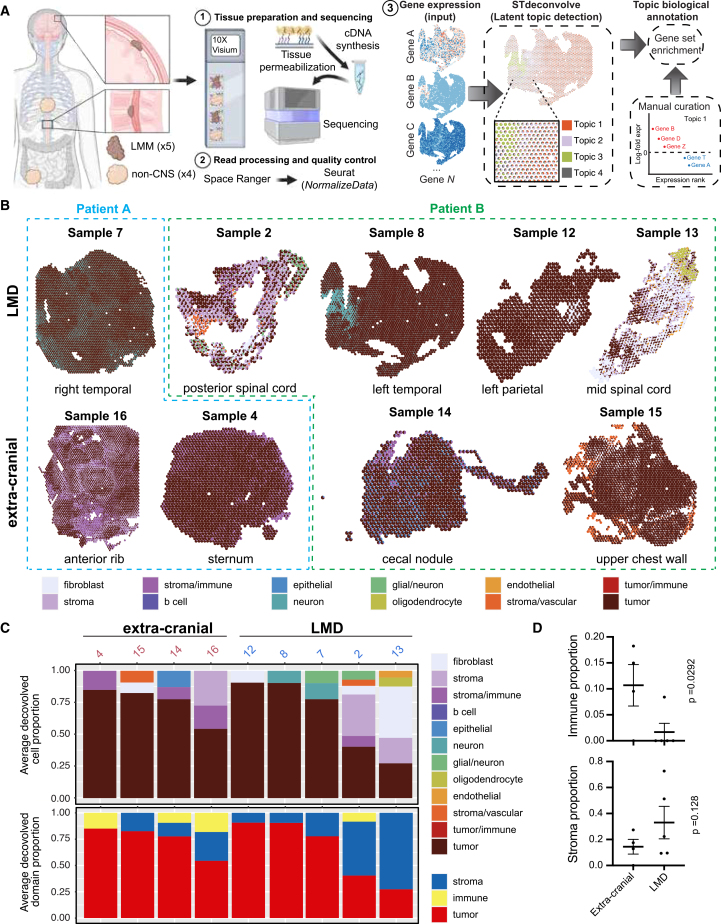
Spatial atlas of melanoma LMD compared with other sites of disease (A) Schematic of the spatial transcriptomic analysis workflow, including tissue selection, preparation, sequencing, data quality control, and cell-type deconvolution analysis. Created with BioRender.com. (B) Tissue maps showing spatial cell-type deconvolution for each spot. The cell-type color key is the same for (B) and (C). (C) Bar graphs showing the average deconvolved cell proportion for the major cell types identified in each sample, organized by tissue of origin. (D) Scatterplots showing the mean proportion of spots predominantly comprised of immune and stromal cells in extra-cranial versus leptomeningeal metastasis samples. Error bars indicate standard error.

To define the cellular landscape of melanoma LMD, we performed an analysis of spatial transcriptomic data from nine tissue specimens from two patients, including patient-matched tissues from leptomeningeal metastases (five tissues) and extra-cranial sites (four tissues) from two melanoma patients with leptomeningeal involvement (Figure 1A; Table S1). The spatial transcriptomics profiling allowed us to capture whole-transcriptome level gene counts using a uniform grid of “spots” containing RNA probes, across a region of 6.5 × 6.5 mm. Differential gene expression analysis of spatial transcriptomics data immediately highlighted striking differences in the ecosystem of the tumor at the leptomeninges and those at extra-cranial sites from the same patients, including the gene expression profiles of the tumor cells themselves (Figures S1A and 1B; Table S2). We identified cell types within each spot using gene set enrichment to delineate tissue niches at different disease sites. Qualitative examination by two expert pathologists of the tissue niches identified in the spatial transcriptomics data indicated agreement with the overall morphology observed in the H&E staining of the same tissues (Figures 1B and S2), hence validating the existence of the tissue niches and enabling downstream comparative analysis among the tissue niches. Our analysis revealed noticeable differences in stromal and immune infiltration between the leptomeninges and extra-cranial sites, as suggested by the number of spots assigned to the stroma and immune tissue niches (Figure 1C). Most extra-cranial samples (3/4) demonstrated some level of immune infiltration, while the majority (4/5) of LMD samples lacked measurable immune infiltration (*p* < 0.05, Figures 1C and 1D). Meanwhile, tumors at the leptomeninges demonstrate a trend for higher stromal involvement instead, although this trend was not statistically significant (*p* = 0.128, Figures 1C and 1D).

### Spatially driven signaling in melanoma LMD

**Figure 2:**
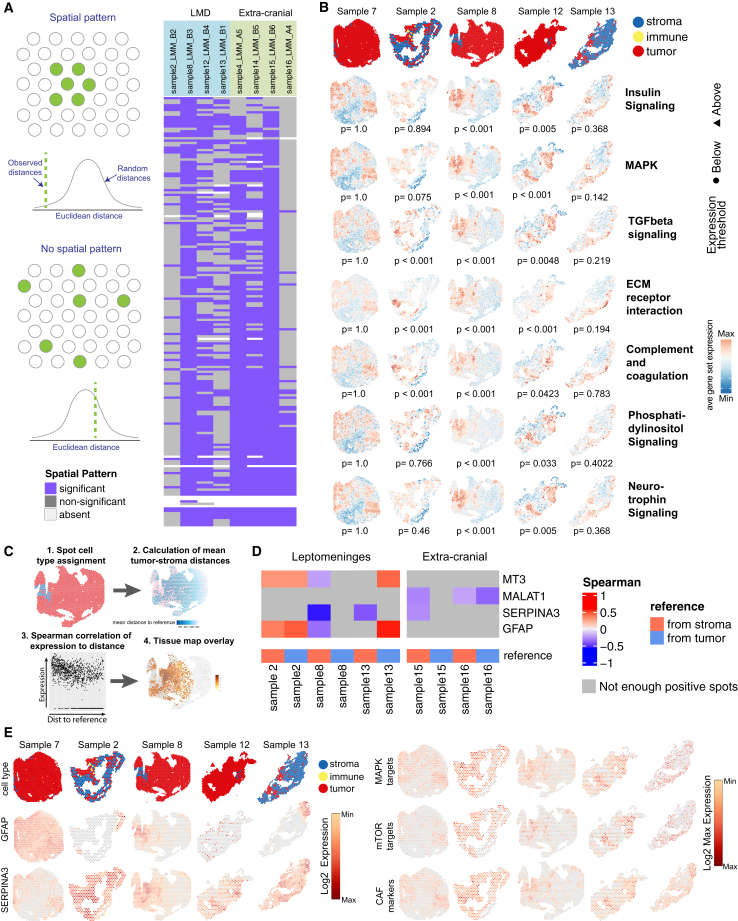
Spatially driven signaling in melanoma LMD (A) Schematic showing the basic principles behind the algorithm used to identify KEGG pathway enrichment that have significant spatial patterns (left) and a heatmap highlighting the quantity and similarities/differences of KEGG pathways showing significant spatial pattern within each tissue sample (right). (B) Spatial tissue maps showing the position of the stroma, immune, and tumor spots along with tissue maps visualizing the average gene set expression for major pathways important in melanoma biology and drug resistance for each LMD sample. (C) A schematic showing the workflow for determining which genes show a correlation between the level of expression and the distance between tumor and stroma spots (see STAR Methods, Gene expression gradients at tissue niche interfaces, for details). (D) A heatmap of the genes showing a significant correlation between expression and the distance between tumor and stroma spots across multiple LMD samples. (E) Spatial tissue maps showing the position of the stroma, immune, and tumor spots along with tissue maps visualizing the log2 gene expression for GFAP and SERPINA3, and the log2 max expression for sets of genes that are MAPK targets, mTOR targets, and CAF markers (Table S4).

To identify potential spatially driven signaling in melanoma LMD, we examined KEGG pathway enrichment that exhibited significant spatial distribution patterns (*p* < 0.05). We defined spatial patterns as areas of high expression with respect to the entire tissue using the STenrich^13^ method (i.e., expression hotspots, see STAR Methods) (Figure 2A). The STenrich *p* values and spatial pathway maps for all analyzed pathways in all samples are included in Table S3 and Data S1. To better visualize regions of the tumor, stroma, and immune infiltration on the spatial maps for consecutive visualizations, we condensed the cell subtype categories into the "tumor," "stroma," and "immune" categories (Figures S3 and S4). Overall, we identified differences in the spatial expression of several pathways in LMD and extra-cranial metastases (Figure 2B; Data S1; Table S3). Notably, we have found spatial patterns of signaling previously associated with LMD biology, such as complement and coagulation and TGF-β signaling (Figure 2B).^5^ Importantly, we have noted upregulation of several pathways critical for melanoma biology and MAPK inhibitor resistance in areas of tumor-stroma interface, including ECM receptor interaction, insulin signaling, MAPK signaling, phosphatidylinositol signaling, and neurotrophin signaling (Figure 2B).^5^^,^^14^^,^^15^^,^^16^^,^^17^^,^^18^^,^^19^^,^^20^

### Tumor-stroma interactions in melanoma LMD

The greater extent of tumor-stroma interfaces and the apparent upregulation of several processes that are integral to melanoma growth and drug resistance in the leptomeningeal tissues prompted us to examine the spatially distinct functional interactions among the melanoma tumor cells and the leptomeningeal stroma. Instead of relying on a subjective determination of a tumor/stroma interface, we developed a novel analysis algorithm called STgradient^13^ (see STAR Methods) that leveraged the spatial distances between tumor and stroma spots and their correlation to the expression of individual genes (Figure 2C). Genes demonstrating a spatial expression gradient are shown in Tables S4 and S5 for all samples. The genes with a significant correlation between the level of expression and the spatial proximity between tumor and stromal spots that are enriched in leptomeningeal tissues (patterns observed in multiple samples) include MT3, SERPINA3, and glial fibrillary acidic protein (GFAP) (Figure 2D). GFAP upregulation is a known marker of glial scarring related to several neurodegenerative conditions.^21^^,^^22^ MT3 is well implicated in several neurodegenerative processes, including cerebral ischemia, Parkinson’s disease, and Alzheimer’s disease.^23^ These findings are consistent with the clinical experience that most patients with LMD develop debilitating multi-focal neurological symptoms, including cranial neuropathies, headaches, ataxia, limb weakness, numbness, pain, or paralysis.^2^^,^^24^^,^^25^^,^^26^ Ischemic infarction and other disruptions caused by increased intracranial pressure due to the impedance of CSF flow may contribute to some of these symptoms. However, the spatial expression gradient we observed in GFAP and MT3 expression, with upregulation at the tumor-stroma interface in leptomeningeal metastases, suggests that neural damage occurs directly at the tumor/stroma interface (Figures 2D and 2E).

Overall, LMD tissues had a higher proportion of non-zero SERPINA3-expressing spots per sample than extra-cranial samples, although this difference was not statistically significant (*p* = 0.206, Figure S5A). Three LMD tissues had a sufficient number of stromal cells to examine the changes in the tumor SERPINA3 expression at increasing distance intervals from the stroma, and all three tissues showed a trend of expression decreasing at intervals further away from the stroma (*p* < 0.001, *p* = 1, and *p* < 0.001 for samples 7, 2, and 8, respectively, Figure S5B). After categorizing the distances of tumor spots to the stroma compartment, we observed that the median expression of SERPINA3 in the leptomeningeal samples was significantly higher (*p* < 0.05) within the 2,000 μM proximal to stroma than at larger distances. Meanwhile, the only extra-cranial metastasis sample with sufficient stromal spots for analysis did not show this trend (*p* = 1, Figure S5B). Re-analysis of our previously published mass-spectrometry-based proteomic profiling of CSF from patients with melanoma LMD shows SERPINA3 to be enriched in patients with LMD compared with no LMD controls (1.21 Log2 ratio, *p* < 0.001).^5^ SERPINA3 regulates the PI3K and MAPK pathways by activating the IGF1R/integrinα5β1 signaling^27^ (Figure S6A). Knowing that melanoma tumors at the leptomeninges rarely respond to MAPK inhibitor therapy and grow rapidly, we examined the spatial expression of MAPK targets and targets of mTOR in the leptomeningeal tissues (Table S6). Not surprisingly, we see a strong upregulation of MAPK and mTOR target expression in the tumor regions at the leptomeninges closer to the tumor-stroma interface (Figure 2E). We noted that the expression of SERPINA3 and IGF1R/integrinα5β1 co-localized at areas of greater stromal interface (Figure S6B), highlighting the potential interaction between the stroma and tumor cells via the SERPINA3 and IGF1R/integrinα5β1 axis. Together, these data suggest a pro-tumorigenic interaction between the tumor and stroma at the leptomeninges.

The tumor cells at the leptomeninges are typically found floating in the CSF fluid or attached to the pia mater tissues on the surface of the brain.^2^ Since the pia mater is comprised predominantly of specialized meningeal fibroblast-like cells,^2^ we set to investigate if these unique cells could become CAFs. Our data demonstrate an enrichment of CAF marker expression (Table S6) at the tumor-stroma interface (Figure 2E).

### Meningeal stroma supports melanoma growth and drug resistance

**Figure 3:**
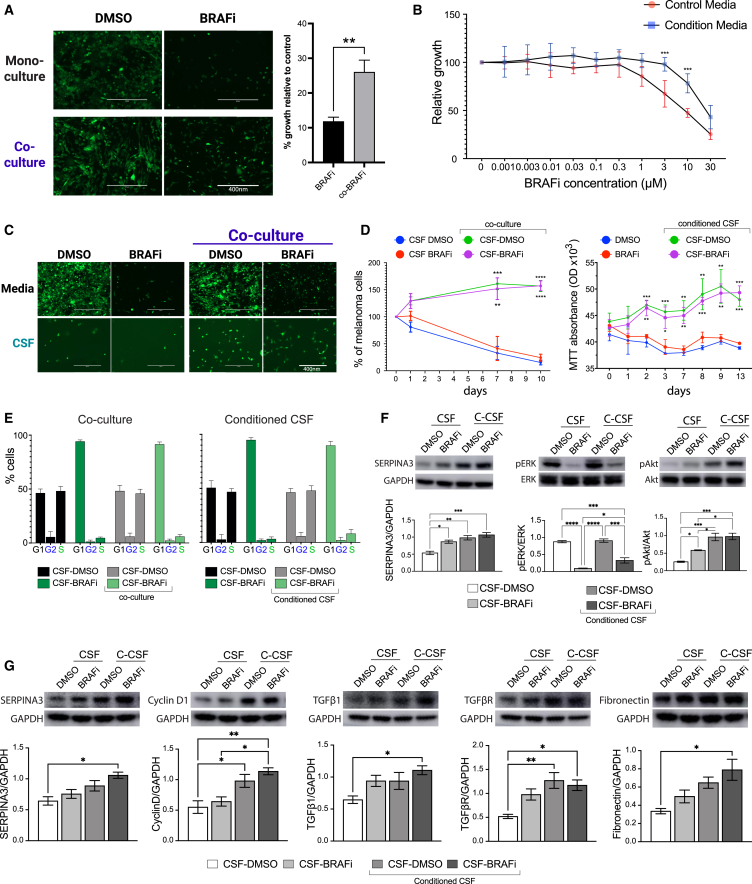
Meningeal cells are required for melanoma survival in the CSF environment (A) Representative microscopy images showing GFP-tagged WM164 melanoma cells treated with 3 μM vemurafenib (BRAFi) or DMSO control in monoculture versus co-culture with primary meningeal cells (left). Bar graphs show the quantification of GFP+ cells across 10 biological replicates relative to the respective vehicle-treated controls (right). Scale bars represent 400 nm. (B) MTT assay showing the relative growth of WM164 melanoma cells treated with increasing doses of vemurafenib (BRAFi) in control medium versus medium conditioned by the co-culture of melanoma with primary meningeal cells. Data represent four independent biological replicates. (C) Representative microscopy images showing GFP-tagged WM164 melanoma cells treated with 3 μM vemurafenib (BRAFi) or DMSO control in monoculture versus co-culture with primary meningeal cells in the context of CSF. Scale bars represent 400 nm. (D) Quantification of the GFP+ WM164 melanoma cells treated with 3 μM vemurafenib (BRAFi) or DMSO control in monoculture versus co-culture with primary meningeal cells for 10 days in CSF (left). WM164 melanoma cells treated with 3 μM vemurafenib (BRAFi) or DMSO control in with regular CSF vs. CSF conditioned by co-culture of melanoma with primary meningeal cells over 13 days (right). Data representative of three biological replicates. Significance was calculated between the co-culture or conditioned medium arm and the respective monoculture control arm. (E) Flow cytometry assessment of cell cycle using PI staining in WM164 melanoma cells treated with 3 μM vemurafenib or DMSO in the context of direct co-culture with primary meningeal cells or with CSF conditioned by co-culture of melanoma with primary meningeal cells. (F) Western blot analysis of WM164 melanoma cells treated with 3 μM vemurafenib or DMSO control in fresh CSF vs. conditioned CSF showing abundance of SERPINA3, pERK(Thr202/Tyr204), ERK, pAKT (Ser473), and AKT. Data represent three independent biological replicates. (G) Western blot analysis of primary meningeal cells stimulated with WM164-conditioned CSF showing the abundance of SERPINA3, cyclin D1, TGF-β1, TGF-βR, and fibronectin. SERPINA3, Cyclin D1 and Fibronectin were blotted on the same membrane and therefore have same loading control. TGF-β1 and TGF-βR were blotted on the same membrane and therefore have same loading control. Data represent three independent biological replicates. All statistical significance was assessed using Student’s t test ∗*p* < 0.05, ∗∗*p* < 0.01, ∗∗∗*p* < 0.001, ∗∗∗∗*p* < 0.0001. Error bars represent standard error.

The majority of melanoma LMD patients do not respond to MAPK-targeting therapy, and we recently showed that CSF from these patients could modulate BRAF inhibitor responses and contribute to drug resistance.^3^^,^^5^ To test whether the meningeal stroma could support melanoma survival in the context of MAPK inhibition, we treated GFP-tagged human melanoma cells with vemurafenib (BRAFi) in monoculture or co-culture with human primary meningeal cells. We noted a significant increase in human melanoma cell survival with human meningeal cell co-cultures (*p* < 0.01, Figure 3A). The same effect was observed in murine melanoma cells with murine primary meningeal cell cultures (Figure S7A). Similarly, the culture of melanoma cells with conditioned medium from melanoma-meningeal co-cultures also demonstrated a protective effect over a range of vemurafenib concentrations in an MTT assay (Figure 3B). Direct co-culture with meningeal cells and stimulation with conditioned medium showed similar levels of protection from MAPK inhibition (Figure S7B). While these experiments were carried out in the context of established normal melanoma cell culture conditions, the leptomeningeal environment is unique because the cells are surrounded by CSF.^3^ Utilizing physiological CSF formulated to mimic the composition of healthy human CSF, we have found that not only do meningeal cells promote MAPK drug resistance but they are also required for melanoma cell survival in CSF. Microscopy images of GFP-positive melanoma cells and MTT assays following treatment in the context of CSF conditioned by melanoma/meningeal cells co-culture (C-CSF) both show profound survival-promoting effects and lack of BRAF inhibitor sensitivity with conditioned CSF (Figures 3C and 3D). We confirmed these findings with an additional melanoma cell line and the BRAF + MEK inhibitor combination using dabrafenib and trametinib (Figures S7C and S7D). Cell-cycle analysis of melanoma cells grown in monoculture and co-culture shows no significant changes in BRAF inhibitor-mediated G1 arrest in the context of either CSF or standard medium, suggesting that the effects are independent of the cell cycle (Figures 3E and S7E). Western blot analysis validated the results observed in the spatial gene expression data, which indicated that co-localized melanoma and primary meningeal cell regions upregulate SERPINA3 when stimulated with co-culture-conditioned CSF (Figures 3F and 3G). Consistent with an increase in MAPK- and PI3K-related signaling observed at the tumor-meningeal stroma interface, we also observed the conditioned CSF to promote recovery of pERK (*p* < 0.05) and amplify pAKT (*p* < 0.05) following BRAF inhibitor treatment (Figure 3F). In meningeal cells, exposure to CSF conditioned by melanoma/meningeal cell co-cultures also promoted cyclin D1, fibronectin, TGF-β1, and TGF-βR expression, suggesting that exposure to tumor cells in the CSF space activates pro-tumorigenic, CAF-associated programs in meningeal stroma (Figure 3G). We observed similar signaling effects under standard complete medium with serum. The basal levels of pERK and pAKT in melanoma cells were much lower when cells were cultured in CSF compared with standard complete medium and, therefore, the meningeal cell-mediated signaling effects are much more pronounced in the CSF co-cultures than under normal medium conditions (Figure S8).

### Meningeal cells promote tumor growth and MAPKi resistance *in vivo*

**Figure 4:**
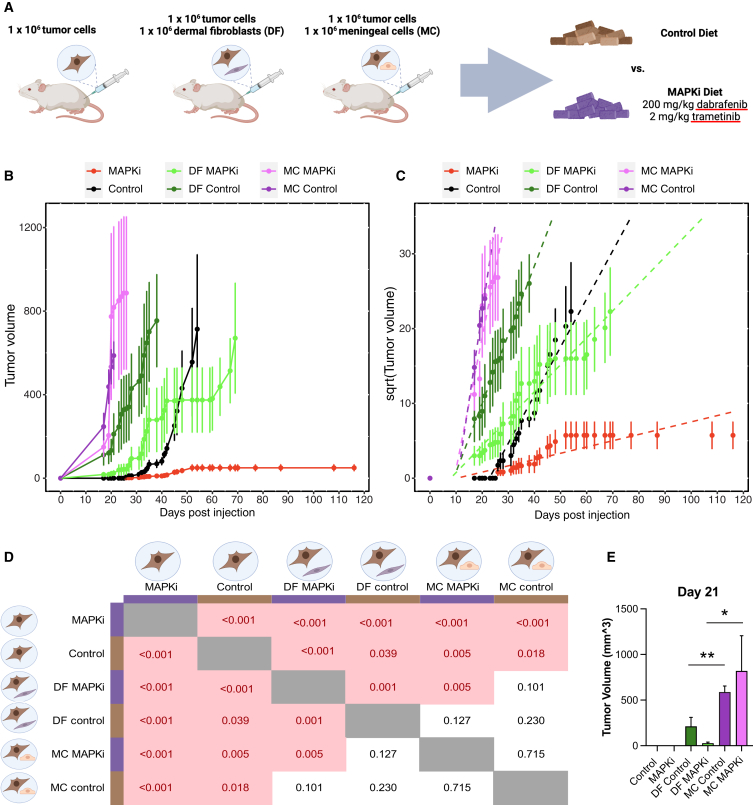
Meningeal cells promote growth and resistance to MAPKi *in vivo* (A) A schematic outlining the experimental cohorts of the animal experiment. Mice were injected subcutaneously with 1 million WM164 cells alone, 1 million WM164 cells with 1 million primary meningeal cells (MCs), or 1 million WM164 cells with 1 million dermal fibroblast (DF) cells. Mice were fed a control rodent diet (control) or a rodent diet formulated with 200 mg/kg dabrafenib and 2 mg/kg trametinib (MAPKi). Created with BioRender.com. (B) Line graph showing the average growth of tumors in each experimental cohort over time, with error bars depicting standard error. Tumor size was fixed following the endpoint of animals until the last animal in the cohort reached the endpoint. (C) Square-root transformation and slope visualization for each experimental cohort in (B). (D) *p* values for each two-way comparison of slope among experimental cohorts (ANCOVA). Icons created with BioRender.com. (E) Bar graph visualizing the average tumor volume in each experimental cohort on day 21. Data represent mean ± standard error for three biological replicates per cohort. Statistical significance was assessed using Student’s t test ∗*p* < 0.05, ∗∗*p* < 0.01. Error bars represent standard error.

We have previously shown that primary human dermal fibroblasts promote melanoma BRAFi resistance.^19^ To compare how well meningeal cells promote tumor growth and drug resistance compared with dermal fibroblasts, we injected human melanoma cells into flanks of animals, either by themselves or in combination with primary dermal fibroblasts or primary meningeal cells in a 1:1 ratio (Figure 4A). Twenty-four hours following implantation, we placed all animals on either control or drug-formulated chow containing the dabrafenib and trametinib combination (MAPKi). As expected, co-injection of melanoma with dermal fibroblasts promoted rapid tumor growth and diminished sensitivity to MAPKi (Figure 4B). Surprisingly, the tumor growth of melanoma cells co-injected with meningeal cells was even faster than those injected with dermal fibroblasts (*p* < 0.01, Figures 4B–4E). These tumors were also even more resistant to MAPKi than those co-injected with dermal fibroblasts (*p* < 0.05, Figures 4B–4E). Pairwise analyses of covariance among linear fit models of each experimental cohort show that the speed of growth (slope) was indeed significantly different among tumor cells injected by themselves, those co-injected with dermal fibroblasts, and those co-injected with meningeal cells (Figures 4C and 4D). At day 21 post-injection, the animals with melanoma cells injected on their own had not started to form measurable tumors yet, while the animals harboring melanoma cells co-injected with meningeal cells were at or approaching the endpoint (Figure 4E).

### Inhibition of tumor-meningeal cell crosstalk abrogates tumor survival and drug resistance

**Figure 5:**
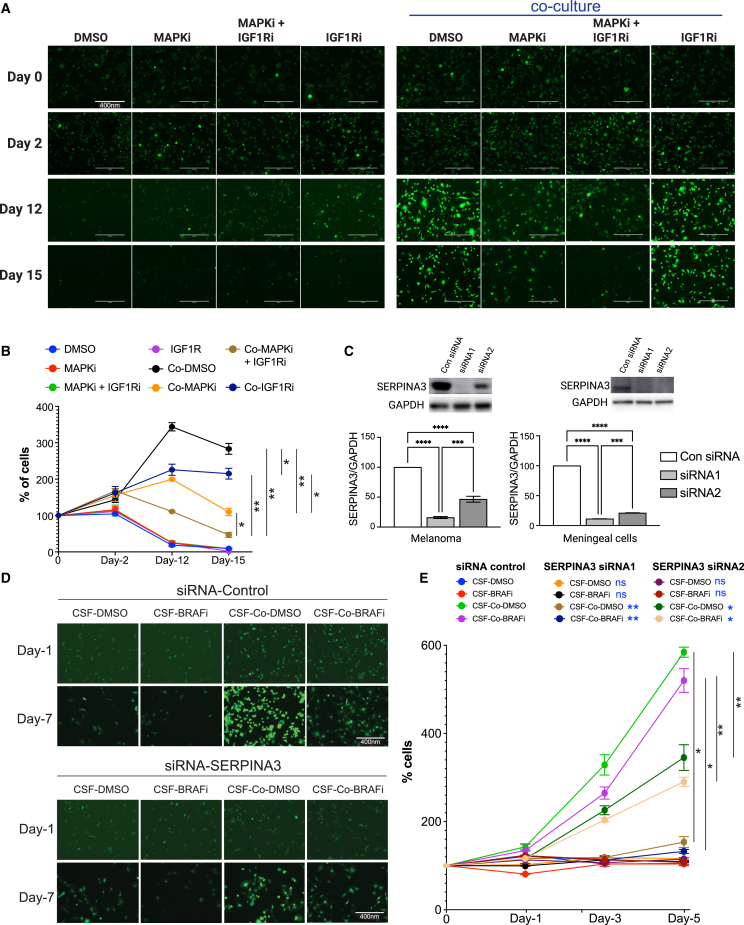
Abrogating SERPINA3/IGF1R signaling sensitized melanoma to MAPKi in the CSF environment (A) Representative microscopy images showing GFP-tagged WM164 melanoma cells treated with DMSO control, 100 nM dabrafenib/10 nM trametinib (MAPKi), 1 μM linsitinib (IGF1Ri), or the triple combination in monoculture versus co-culture with primary meningeal cells in the context of CSF. Scale bars represent 400 nm. (B) Quantification of the GFP+ WM164 melanoma cells treated with DMSO control, 100 nM dabrafenib/10 nM trametinib (MAPKi), 1 μM linsitinib (IGF1Ri), or the triple combination in monoculture versus co-culture with primary meningeal cells for 15 days in CSF. Data representative of two biological replicates. (C) Western blots showing knockdown of SERPINA3 with two individual SERPINA3 siRNAs (siRNA1 and siRNA2) in the WM164 melanoma cells and the HMC meningeal cells. Scrambled sequence siRNA was used as control (Con siRNA). (D) Representative microscopy images showing GFP-tagged WM164 melanoma cells treated with 3 μM vemurafenib (BRAFi) or DMSO (control) in monoculture versus co-culture (Co) with primary meningeal cells in the context of CSF following knockdown of SERPINA3 (siRNA1 and siRNA2) or control siRNA (Con siRNA) in both cell types. Scale bars represent 400 nm. (E) Quantification of GFP-tagged WM164 cells from (D). Data represent two biological replicates. Statistical significance was assessed using Student’s t tests (B, C, and E). Significance is denoted as ∗*p* < 0.05, ∗∗*p* < 0.01, ∗∗∗*p* < 0.001, ∗∗∗∗*p* < 0.0001; ns, not significant. Error bars represent standard error.

Since we observed amplification of AKT phosphorylation during MAPK inhibitor treatment in the context of conditioned CSF, and it has been shown that SERPINA3 promotes AKT signaling via activation of IGF1R,^27^ we tested to see if inhibition of IGF1R would re-sensitize cells to MAPK inhibitor therapy. We noted that the meningeal cultures protected melanoma tumor cells similarly in the context of either single-agent BRAF inhibitor or dual BRAF/MEK inhibitor therapy, with little difference observed in the magnitude of viability rescue between the two therapies (*p* = 0.2722, Figures 3A, S7C, and S7D). Inhibition of IGF1R using linsitinib successfully blocked the survival-promoting effects of meningeal cell co-culture in CSF (Figures 5A and 5B). The MAPK inhibitor resistance effects were also blocked under standard complete medium conditions (Figure S9). We then tested whether SERPINA3 is required for the survival-promoting effects of meningeal cell co-culture using siRNA-mediated knockdown of SERPINA3 (Figure 5C). Knockdown of SERPINA3 significantly reduced melanoma cell growth and MAPK inhibitor resistance in CSF (*p* < 0.01 for siRNA1 and *p* < 0.05 for siRNA2, Figures 5D and 5E). Once again, the MAPK inhibitor resistance effects were blocked during siRNA-mediated knockdown under normal complete medium conditions (Figures S10A and S10B).

## Discussion

Although first described a century and a half ago,^28^ little progress has been made in our understanding of the biology, aggressive nature, and vulnerabilities of LMD.^1^ Most recently, technological advancements such as single-cell RNA sequencing have finally allowed us to glimpse the biology of this difficult-to-sample site.^6^^,^^29^^,^^30^ Although these methods uncovered valuable information, the tumor microenvironment of the cells suspended in CSF may not reflect the tumor microenvironment of the melanoma tumors adherent to the leptomeningeal tissues and the potential interactions occurring among those elements of the tumor microenvironment. It is also becoming increasingly clear that the spatial context of the tumor microenvironment is critical in understanding the functional heterogeneity of the tumor and the tumor’s interactions within the LMD ecosystem. We had previously observed that the CSF of patients with melanoma LMD harbored much fewer immune infiltrates, but the analysis of the CSF component did not provide any insights into the stromal compartment of the tumor microenvironment. Here, we utilized spatial transcriptomics technologies and applied novel analyses^31^ to show that the melanoma tumors at the leptomeninges demonstrate a greater interaction with the stroma than those at extra-cranial sites of disease. We confirm that gene expression involved in pathways that are integral for melanoma growth and drug resistance, including MAPK and phosphatidylinositol signaling,^20^^,^^32^^,^^33^^,^^34^^,^^35^^,^^36^ and processes that we have previously identified in CSF fluid to be important for melanoma LMD, such as TGF-β signaling and complement and coagulation,^5^^,^^37^ are not distributed randomly throughout the tissue but are rather correlated to the proximity of the tumor to the stroma. Instead of relying on a more arbitrary determination of the tumor/stroma interface as has been done in other spatial transcriptomics studies,^38^^,^^39^^,^^40^^,^^41^ we have developed a novel algorithm for calculating the correlation of gene expression to the distance between spots containing tumor and stromal cells, allowing the data to ascertain if areas of high expression occur at the interface between the tumor and stromal cells.

The initial impression of the leptomeningeal environment is that it should be inhospitable to rapidly growing tumor cells. Typically, CSF contains few nutrients or proteins, suggesting few growth factors and metabolites to sustain tumor survival and growth. However, the rapid disease progression of melanoma LMD in most patients suggests otherwise. The stromal component of the leptomeningeal tissues is very unique, comprising of vasculature and specialized fibroblast-like meningeal cells.^8^ Our data show that these meningeal cells turn on pro-tumorigenic signaling when exposed to melanoma cells in the CSF environment and support tumor survival and drug resistance in this otherwise hostile environment. Many previous studies have demonstrated pro-tumorigenic roles of CAFs in melanoma tumors, generally utilizing dermal fibroblast cultures.^19^^,^^42^^,^^43^^,^^44^ In the central nervous system microenvironment, astrocytes have been implicated in aiding melanoma cell metastasis and survival.^45^^,^^46^^,^^47^ We now demonstrate that the meningeal stroma is required for melanoma cells to survive in the CSF environment through upregulation of SERPINA3 expression and subsequent activation of the PI3K/AKT and MAPK signaling. The role of SERPINA3 in regulating the PI3K/AKT and ERK signaling was initially identified in adipogenesis, where SERPINA3 was shown to modulate IGF1R and integrin signaling by inhibiting serine proteases.^27^ Although a great number of intrinsic and acquired MAPK inhibitor resistance mechanisms have now been identified, many of them converge on either the reactivation of ERK signaling or the activation of compensatory pathways through PI3K/AKT.^32^ Here, we show that meningeal cell-induced SERPINA3 expression at the tumor/stroma interface promotes the amplification of PI3K/AKT signaling and the reactivation of ERK in the tumor cells, thereby overcoming BRAF/MEK inhibition. We further demonstrate stronger tumor-promoting effects from co-injection of melanoma cells with meningeal stromal cells compared with dermal fibroblasts. The etiology of these effects is not immediately known but is possibly related to the need to overcome a harsh, nutrient-poor, hypoxic microenvironment found in the leptomeninges. It has previously been demonstrated that fibroblasts from various tissues differ greatly in their transcriptional profiles, with less than a 20% similarity among those found in the mouse heart, skeletal muscle, intestine, and bladder.^48^ It is also worth noting that, unlike dermal fibroblasts, both melanocytes and meningeal fibroblast-like stroma are derived from the neural crest.^49^ It is possible that the common developmental origins of both the melanocytes and meningeal cells facilitate the synergy in their interactions. However, we have not ruled out the possibility that similar interactions occur at other sites of metastasis.

Most patients with melanoma LMD do not respond to MAPK-targeting therapies such as the BRAF/MEK inhibitor combination.^1^ Our data identify an LMD-specific vulnerability in melanoma cells, which can be exploited therapeutically to improve sensitivity to MAPK-targeting therapies. Some of the major difficulties faced by patients with melanoma LMD are the severe neurological symptoms that negatively impact their quality of life, including pain, nausea, diplopia, weakness, and paralysis.^1^^,^^2^ These symptoms have primarily been attributed to the development of hydrocephalus and increased intracranial pressure due to the tumor obstructing normal CSF flow.^50^^,^^51^ However, our spatial analysis of patient specimens shows increased MT3 expression and expression of the reactive gliosis marker GFAP at the tumor/stroma interface. Previously, GFAP and MT3 have been linked to trauma, ischemia, and neurodegenerative diseases, including encephalomyelitis, multiple sclerosis, Parkinson’s disease, Alexander disease, Alzheimer’s disease, and amyotrophic lateral sclerosis.^21^^,^^23^^,^^52^^,^^53^^,^^54^ Our data provide evidence supporting a direct tumor-mediated reactive gliosis and possible neuronal damage occurring at the cellular level. These data are in line with the observations that interventions relieving hydrocephalus, such as ventroperitoneal or lumbar shunting, can result in a reduction of some neurological symptoms such as headache and nausea but may have no effect on other symptoms such as cognitive dysfunction or gait dysfunction.^50^

One limitation of our work is that we initially profiled relatively few tissues from few patients (nine tissues total from two patients), limiting the power of our analysis. It is not entirely clear if all melanoma LMD tumors will exhibit upregulation of SERPINA3, or whether past treatment history may have affected our results as the majority of progressing late-stage melanoma patients are heavily pre-treated and we did not have enough patients on this study to draw any treatment-related conclusions. In addition, most of the leptomeningeal samples were from one patient (one LMD tissue from patient A and four LMD tissues from patient B), therefore it is possible that our findings were driven by the data from patient B. However, we did observe consistency in the spatial landscape, signaling, and tumor-stroma interactions in LMD tissues between the two patients, especially between the two LMD tissues harvested from the temporal region. The rarity of this patient population (5%–7% of metastatic melanoma patients), the sizable gaps in our understanding of melanoma biology at the leptomeninges, and the preclinical *in vitro* and *in vivo* validation of our findings highlight the unparalleled value of this work. Furthermore, several small-molecule and antibody-based inhibitors of IGF1R and the PI3K/AKT/mTOR pathway are currently being tested in the clinic,^55^^,^^56^^,^^57^^,^^58^ highlighting the potential translatability of combining these drugs with BRAF/MEK inhibitor therapy for the treatment of melanoma LMD. It is also important to note that the spatial transcriptomic approach utilized here is at the resolution of 55-μm spots, which may include more than one cell and more than one cell type, requiring computational deconvolution methods. One potential limitation of LDA-based deconvolution is that accuracy decreases when the differences in gene expression across the tissue are not shallow, as is the case for tissues with homogeneous spatial composition.^59^ However, the LMM tissue microenvironment is varied in cell types, and spatial gene expression differences were evident (see gene expression gradient analysis, for example). Another limitation of LDA models is that zero-excess in gene expression could reduce the power to detect heterogeneous topics.^59^ The excess of zeroes in gene expression is commonplace in ST experiments. Hence, our niche-level analysis was performed as a workaround to the potential low accuracy to detect differences among spots, especially for cell types with low abundance.

These results may have wider implications on the biology of LMD from other tumor types, including breast cancer, lung cancer, and lymphoma. All of these tumor types have been shown to have tumor-promoting interactions with fibroblast-like stroma,^60^^,^^61^^,^^62^^,^^63^^,^^64^^,^^65^^,^^66^^,^^67^ and all of these tumor types seed to the leptomeninges.^68^^,^^69^ Due to the unique biology and relatively harsh conditions of the leptomeningeal tumor microenvironment, it is likely that other tumor types also rely on tumor-promoting interactions with the meningeal stroma. Therefore, developing strategies for targeting these interactions may be of high value for LMD arising from multiple cancer types.

### Limitations of the study

The number of samples profiled in the spatial transcriptomic analysis is quite few (nine tissues from two patients), limiting the power of our analysis. Therefore, it is possible that the SERPINA3 findings from this cohort may not represent the biology of all melanoma patients with LMD. There are also limitations to using LDA-based deconvolution methods for spatial transcriptomics analysis. There is a reduction of accuracy when the variations in gene expression across tissue are not shallow and there can be a reduction in power to detect heterogeneous topics for datasets with an excess of zeros.

## STAR★Methods

### Key resources table

**Table:** 

REAGENT or RESOURCE	SOURCE	IDENTIFIER
**Antibodies**		

SERPINA3	Genetex	GTX55503
pERK	Cell Signaling Technology	4370S; RRID:AB_2315112
ERK	Cell Signaling Technology	4695S; RRID:AB_390779
pAKT	Cell Signaling Technology	4060S; RRID:AB_2315049
AKT	Cell Signaling Technology	9272S; RRID:AB_329827
Cyclin D1	Cell Signaling Technology	55506S; RRID:AB_2827374
Fibronectin	BD	610077; RRID:AB_2105706
GAPDH	Sigma Aldrich	G8795; RRID:AB_1078991

**Biological samples**		

9 tissue samples from patients	Postmortem collection at Moffitt Cancer Center	N/A

**Chemicals, peptides, and recombinant proteins**		

vemurafenib	SelleckChem	S1267
dabrafenib	TargetMol	T1903
trametinib	TargetMol	T2125
linsitinib	TargetMol	T6017
MTT	Sigma	

**Critical commercial assays**		

Visium Spatial Gene Expression Starter Kit	10X Genomics	1000200

**Deposited data**		

Spatial Transcriptomics Data	This manuscript	GEO: GSE250636

**Experimental models: Cell lines**		

WM164 melanoma cell line	Wistar Institute	WM164
SM1 melanoma cell line	UCLA	SM1
Human Meningeal cells	Sciencell	1400
Mouse meningeal cells	Moffitt C57 mice	N/A

**Experimental models: Organisms/strains**		

BALB SCID mice	The Jackson Laboratory	001803

**Oligonucleotides**		

SERPINA3 siRNA 1		
SERPINA3 siRNA 2		
Control siRNA		

**Software and algorithms**		

Spatial Transcriptomics Analysis Code	This manuscript	oospina/spatial_transcriptomics_leptomeningeal_disease

**Other**		

Meningeal cell medium	Sciencell	1401
Synthetic Cerebrospinal Fluid	Ecocyte Bioscience	LRE-S-LSG-1000-1

### Resource availability

#### Lead contact

• Further information and requests for resources and reagents should be directed to and will be fulfilled by the lead contact, Inna Smalley (Inna.Smalley@moffitt.org).

#### Materials availability

• This study did not generate new unique reagents.

#### Data and code availability

• Spatial transcriptomics data have been deposited at GEO and are publicly available as of the date of publication. Accession numbers are listed in the key resources table.
• All original code has been deposited at GitHub and is publicly available as of the date of publication. Identifier is listed in the key resources table.
• Any additional information required to reanalyze the data reported in this paper is available from the lead contact upon request.

### Experimental model and study participant details

#### Human participants

This study was conducted in accordance with recognized ethical guidelines (e.g., Declaration of Helsinki, CIOMS, Belmont Report, U.S. Common Rule) and appropriate institutional approvals. Nine human melanoma specimens from two patients were procured postmortem under protocols approved by the Institutional Review Board (MCC 21044 and MCC 20779).

#### Cell lines

WM164 established human melanoma cell line isolated from a male patient (generously shared by Dr. Meenhard Herlyn at the Wistar Institute), SM1 established murine melanoma cell line from isolated from a female mouse (generously shared by Dr. Antony Ribas, UCLA), primary Human Meningeal Cells (HMC, Scincell, catalog # 1400, gender not provided by source) and primary murine meningeal cells (isolated from female C57BL6J mice in our lab) were utilized. The identities of all cell lines were confirmed through short tandem repeat validation analysis, and all cell cultures were routinely tested for mycoplasma contamination. Melanoma cell lines were maintained in 5% FBS/RPMI-1640, and primary meningeal cells were maintained in a Meningeal Cell Medium (Sciencell, catalog # 1401). Assays in this manuscript were performed in either standard medium or cerebrospinal fluid (CSF) obtained from Ecocyte bioscience (LRE-S-LSG-1000-1) supplemented with 1% BSA.

#### Animals

All mouse work was conducted in accordance with recognized ethical guidelines and IACUC approval. BALB SCID female mice approximately 12 weeks of age were used to evaluate the effect of HMC on the development of melanoma tumors. Mice were injected subcutaneously with 1 million WM164 cells alone, 1 million WM164 cells with 1 million primary meningeal cells (MC), or 1 million WM164 cells with 1 million dermal fibroblast (DF) cells. Once the tumors were palpable (on day 17), animals were randomized into treatment groups using an online randomization calculator from GraphPad and started on either a control rodent diet or a rodent diet formulated with 200 mg/kg dabrafenib and 2 mg/kg trametinib (MAPKi) as previously described (Research Diets).^70^

### Method details

#### Spatial transcriptomics

Frozen tissue specimens were processed according to the Visium Spatial Gene Expression User Guide using reagents from the Visium Spatial Gene Expression Kit (10X Genomics, Pleasanton, CA). The Visium Spatial Gene Expression assay allows the profiling of tissue slices no larger than 6.5 x 6.5 mm. Within each 6.5 × 6.5 mm, 5000 55-micron spots containing the RNA probes are evenly spaced with 100 microns separating the centers of each two spots. The uniform grid of this assay allows us to sample the entire tissue slice in a spatially unbiased manner (i.e., RNA is quantified across the entire tissue).

#### Spatial transcriptomics analysis

Sequence.fastq files were generated using the default settings with *spaceranger mkfastq* from base call intensities outputs from the two Illumina NextSeq 2000 runs. Then, the reads were passed to *spaceranger count* along with H&E.tif images from each sample. We used the automatic alignment functionality in *spaceranger count* to spatially register the spots to the H&E image pixels. In cases where the tissues featured holes or tears and noise background counts were retrieved from those areas without tissue, we used 10X Loupe v4 to align the spots to the tissue images manually and passed the resulting.json file to *spaceranger count* via the *--loupe-alignment* option. The *spaceranger count* generated BAM files with aligned reads to the h38 human genome, as well as sparse read count matrices containing the number of RNA counts for each gene at each spot and associated spot coordinates. All spatial RNA-seq datasets will be uploaded to GEO and made publicly available upon publication. All code utilized for analysis is uploaded to GitHub and will be made publicly available upon publication.

#### Identification of cell types and tissue niches (gene expression deconvolution)

Given that Visium spots are multicellular and may contain multiple cell types, we used we used Latent Dirichlet Allocation (LDA), as implemented in the R package STdeconvolve,^59^ to infer the cell type composition within each spot. The algorithm detects latent topics in the gene expression data. The latent topics represent transcriptional profiles of cell types potentially present in the spots. To infer the number of topics (*K*) present in each ST sample, we ran the algorithm from *K* = 3 to *K* = 15. Identification of the *K* value with the best fit to the data was done by examining the LDA model’s perplexity and the number of topics with low representation in the spots. We then selected the *K* value corresponding to the model with the lowest perplexity and the lowest number of low-abundance topics (Figure S11). To assign a biological identity to the topics, we applied gene set enrichment analysis (GSEA) on each topic gene expression (*annotateCellTypesGSEA* function). The cell type gene markers identifying cell types were extracted from the ENCODE and BluePrint databases^71^^,^^72^ using the R package celldex.^73^ Manual curation of topic biological identities was done based on examination of the genes for each cell type compared to the average expression across all cell types. Genes with log-fold >1 for a given cell type were selected as markers. We manually annotated cell types by examining the log-fold gene expression of each topic of the top 15 genes with the highest log-fold change and the bottom 15 genes with the lowest log-fold change in each topic, compared to the average expression of those same genes across all other topics. The changes made using the manual curation procedure are presented in the supplemental materials (Table S7). Given that cell type heterogeneity can be obscured by the multicellularity of spots, we also assigned each spot one of three categories (tumor, stroma, immune) for other downstream analyses (see *Gene expression gradients at tissue niche interfaces* for details).

#### Spatial pathway enrichment analysis

We aimed to identify gene sets that showed evidence of spatial expression "hotspots." We modified the *STenrich* function from the R package spatialGE^13^^,^^74^ to test for hotspots in the average expression of KEGG pathway genes. The method is a permutation-based adaptation of a previously published method.^38^ The algorithm identifies spots with average log-normalized gene set expression above the average plus one standard deviation across the entire tissue. Next, Euclidean distances among the high-expression spots are calculated, an equal number of spots is randomly chosen from the entire tissue, and Euclidean distances among the randomly selected spots are calculated. The random selection procedure was conducted 1000 times. Finally, the sum of distances among the high expression spots was compared against the 1000 sums of distances from the randomly selected spots (null distribution). If the sum of distances among high expression spots was higher than the null distribution, it indicated that the spots expressing the pathway are closer to each other than expected by chance (i.e., spots are aggregated in a hotspot).

#### Gene expression gradients at tissue niche interfaces

The spatial patterns of gene expression at the interface of two niches are often studied by performing differential gene expression analysis on clusters that lie between the niches. Here, we opted for an alternative approach to study gradients of gene expression, hence overcoming the necessity of defining an interface cluster (Figure 2C). We have called this approach STgradient,^13^

The algorithm is as follows and is depicted in Figure S12.

(1) Starting from the cell types identified with STdeconvolve, we determined the most abundant cell type within each spot (cell type proportion ≥50%).
(2) Using this dominant cell type for each spot, the spots were then classified as either in the “tumor”, “immune”, or “stroma” compartments of the tissue.
  a. Spots classified as “tumor” contained the highest proportion of melanoma cells.
  b. Spots classified as “immune” showed a B cell or “stroma immune” signature.
  c. Spots that did not fall in the previous two categories (i.e., “tumor” or “immune”) were assigned to “stroma” category.
(3) Next, we calculated the Euclidean distances of each spot not in the reference niche to the reference niche. For example, if the reference niche was the “tumor” niche, we computed the distrance for each spot not in the “tumor” niche to the “tumor” niche. This was done for both the setting where the reference niche was the “tumor” or the “stroma”. In each scenario, a Euclidean distance matrix was generated containing the Euclidean distance of each spot from the reference niche.
(4) For each spot not contained within the reference region, the average distance to the spots in the reference niche was calculated (Figure 2C). The result was a single average distance value for each spot in the non-reference niche to the reference niche.

Spearman’s correlation coefficients were calculated between the average distances and log-transformed normalized gene expression using the spots not within the reference region. The p-values associated with the correlation coefficients were used to ascertain whether a given gene exhibited an expression gradient (i.e., the expression changed as a function of distance from the reference niche).

#### *In vitro* cell growth/viability analysis

Human melanoma (WM164) and mouse melanoma (SM1) cell lines were plated on cell culture plates as a monolayer or in co-culture with human meningeal cells (HMC) or primary mice meningeal cells (MMC). After 24 h, all plates were treated with either BRAF inhibitor (vemurafenib 3 μM monotherapy or trametinib 10nM plus dabrafenib 100nM combination), BRAF inhibitor plus insulin growth factor 1 inhibitor (linsitinib 1μM) or vehicle (DMSO). The number of the GFP-tagged cells was quantified using ImageJ software.

#### Conditioned medium and CSF

Conditioned media was generated by adding 10 mL of meningeal medium or CSF to a co-culture of WM164 plus HMC or SM1 plus primary mice meningeal cells in 10-cm culture plates. After 24 h of incubation, the medium/CSF was removed and centrifuged at 25000 rcf for 5 min at room temperature to remove any cell debris. The medium was filtered through a 0.22-micron filter before mixing with fresh meningeal medium or fresh CSF at a ratio of 1:1.

#### MTT assays

MTT assays were carried out as described previously.^75^ Briefly, one thousand WM164 cells were cultured in each well of a 96-well plate in either fresh or conditioned CSF. The next day, the cells were exposed to different concentrations of vemurafenib (0–30 mM). The plate was incubated at 37°C and 5% CO^2^ for three days. On day 4, 20uL of MTT reagent was added to each well and incubated for 3 h, then CSF was removed, and MTT crystals were solubilized in 50μL of DMSO. Absorbance was measured at 490 nm.

#### Cell cycle assays

Cell cycle analysis was carried out as described previously.^75^ Cells were plated in a 10-cm culture plate and allowed to grow to 70% confluency. Cells were harvested and washed with 2 mL of phosphate-buffered saline (PBS). The cell pellet was re-suspended with 2 mL ice-cold 70% ethanol and fixed overnight. The next day, the cells were rehydrated with 20 mL PBS for 30 min at room temperature and then centrifuged and washed with PBS. Cells were stained with propidium iodide. Data was acquired using the FACSCanto (BD) instrument and analyzed using ModFit software.

#### Western blots

Western blot analysis was carried out as described previously.^20^ Protein extracts were immunoblotted using antibodies against SERPINA3 (1:1000, Genetex), phospho-ERK (Thr202/Tyr204, 1:1000, Cell Signaling Technology), ERK(1:1000, Cell Signaling Technology), phospho-Akt (1:1000, Cell Signaling Technology), Akt (Ser473, 1:1000, Cell Signaling Technology), CyclinD1 (1:1000, Cell Signaling Technology), TGF β1(1:1000, Cell Signaling Technology), Fibronectin (1:1000, BD) and GAPDH (1:5000, Sigma Aldrich). Digitalized blot images were developed using Amersham Imager 600 (Amersham Bioscience). Quantification of the protein expression was performed using ImageJ software.

#### siRNA knockdowns

Electroporation-assisted siRNA knockdown of SERPINA3 (or scrambled control) was carried out using the Neon transfection system 100 μL kit following the manufacturer’s protocol (Invitrogen). Electroporation was performed at a pulse voltage of 1200. Cells were then seeded in 10 cm cell culture plates containing pre-warmed medium. SERPINA3 knockdown was confirmed by Western Blot.

### Quantification and statistical analysis

Western blot data was analyzed by GraphPad Prism using ordinary one-way ANOVA followed by Tukey’s multiple comparison test. Cell counting was analyzed using an unpaired parametric student’s T-test. *p*-values were considered significant if less than 0.05. Significance is annotated on figures as follows: ∗*p* < 0.05, ∗∗*p* < 0.01, ∗∗∗*p* < 0.001, ∗∗∗∗*p* < 0.0001.

## Supplemental information

Document S1. Figures S1–S12 and Tables S1 and S6

Table S2. Differentially expressed genes among tissue types (LMM vs. extra-cranial) inferred with Stdeconvolve, related to Figure 2 Differential expression tests were conducted using Seurat using the FindAllMarkers function.

Table S3. Adjusted *p* values for KEGG pathway spatial analysis, related to Figure 2 The test only proceeded if at least 20 spots over the high expression threshold were present in the sample, with a threshold of mean expression +1 standard deviation.

Table S4. Summary Spearman coefficients for all samples, related to Figure 2

Table S5. Summary Spearman coefficients for LMD, related to Figure 2

Table S7. MANUAL CURATION, related to Figure 1 Changes made to the automatic GSEA-based classification of the spots in each of the Visium samples. Topic refers to the STdeconvolve topic on which GSEA was conducted. The GSEA_cell_type column includes the classification assigned using the annotateCellTypesGSEA function from STdeconvolve. For each topic, the genes with highest log-fold change compared with the rest of the topics were identified, which we used to re-assign or re-name each topic (Change_based_on_genes). The Curated_cell_type column contains the final annotation for each topic resulting from manual curation. Each tab/panel of the table pertains to a different sample: (A) sample2_LMM_B2, (B) sample4_LMM_A5, (C) sample7_LMM_A1, (D) sample8_LMM_B3, (E) sample12_LMM_B4, (F) sample13_LMM_B1, (G) sample14_LMM_B5, (H) sample15_LMM_B6, (I) sample16_LMM_A4.

Data S1. STenrich *p* values and spatial pathway maps for all analyzed pathways in all samples, related to Figure 2

Document S2. Article plus supplemental information
