# Supplementary material for: Spatial transcriptomics analysis identifies a tumor-promoting function of the meningeal stroma in melanoma leptomeningeal disease
Source: Cell Rep Med. 2024 Jun 11;5(6):101606. doi: 10.1016/j.xcrm.2024.101606 (PMC11228800; doi:10.1016/j.xcrm.2024.101606)
Supplement: Document S1. Figures S1–S12 and Tables S1 and S6 [file mmc1.pdf]

Cell Reports Medicine, Volume *5*

Supplemental information

Spatial transcriptomics analysis identifies a tumor-promoting function of the meningeal stroma in melanoma leptomeningeal disease

Hasan Alhaddad, Oscar E. Ospina, Mariam Lotfy Khaled, Yuan Ren, Ethan Vallebuona, Mohammad Baraa Boozo, Peter A. Forsyth, Yolanda Pina, Robert Macaulay, Vincent Law, Kenneth Y. Tsai, W. Douglas Cress, Brooke Fridley, and Inna Smalley

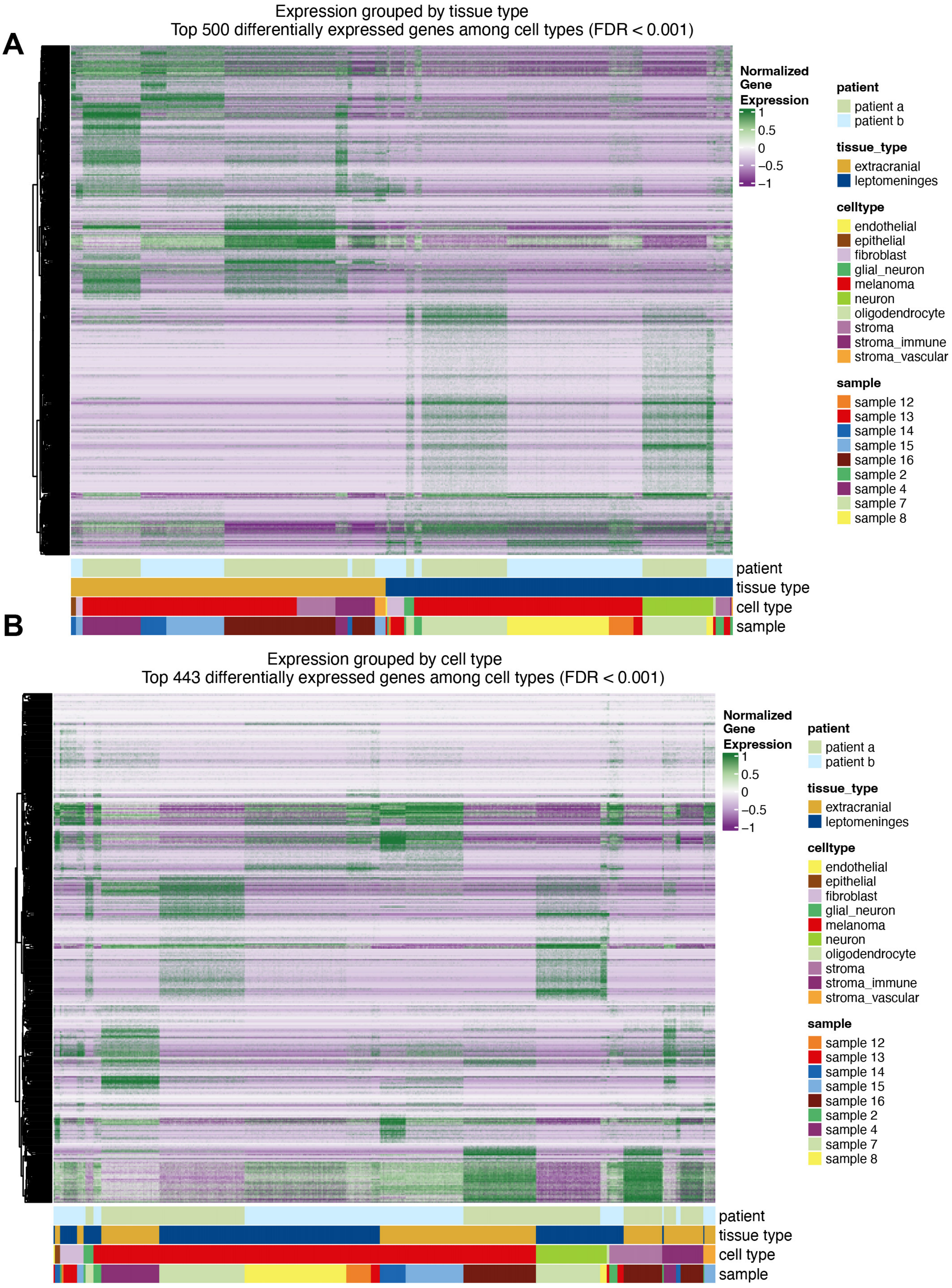

Supplemental Figure 1. Related to Figure 1. **A.** Heatmap of the differentially expressed genes in spots grouped by tissue type **B.** Heatmap of the differentially expressed genes in spots grouped by cell type type.

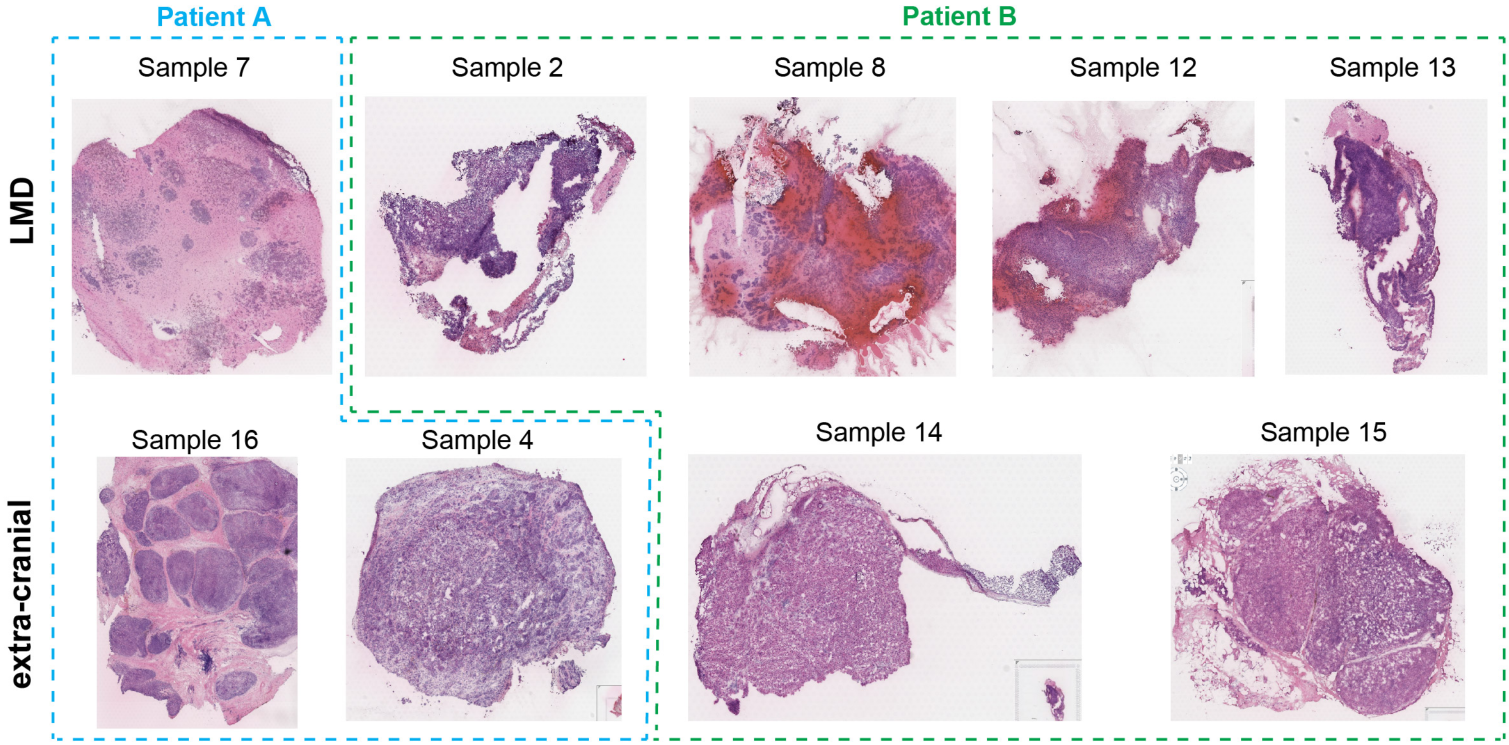

Supplemental Figure 2. Related to Figure 1. H & E images for the melanoma leptomeningeal metastasis samples and patient-matched extra-cranial metastasis samples matching the cell type deconvolution in Figure 1B.

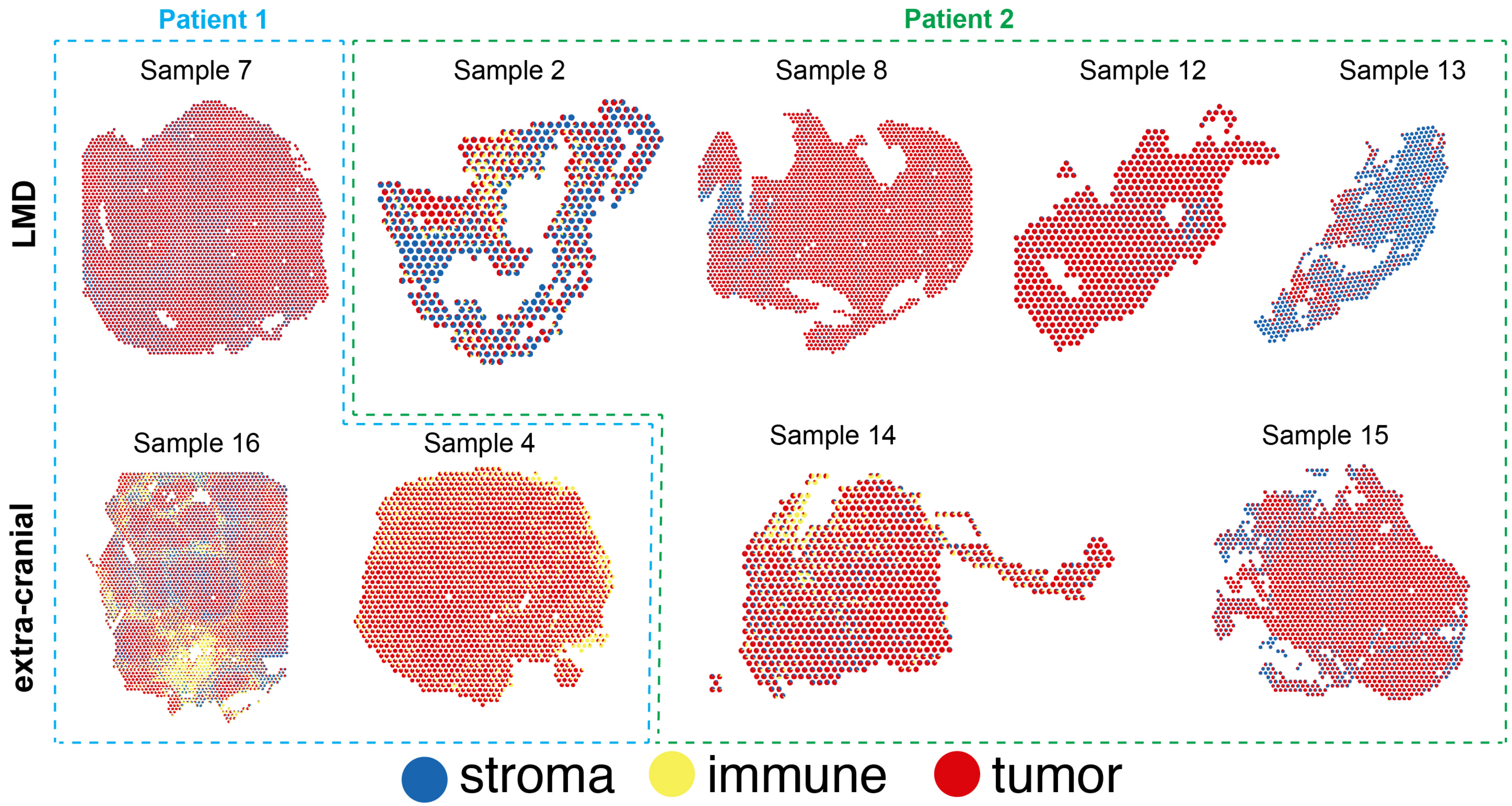

Supplemental Figure 3. Related to Figure 2. Spatial tissue maps showing the deconvolution of the stroma, immune, and tumor cell types within each spot. To better visualize regions of the tumor, stroma and immune infiltration on the spatial maps for consecutive visualizations, the cell subtype categories were condensed into the "tumor", "stroma" and "immune" categories. Each spot was divided into piecharts for the proportion of the stroma, immune or tumor cells present.

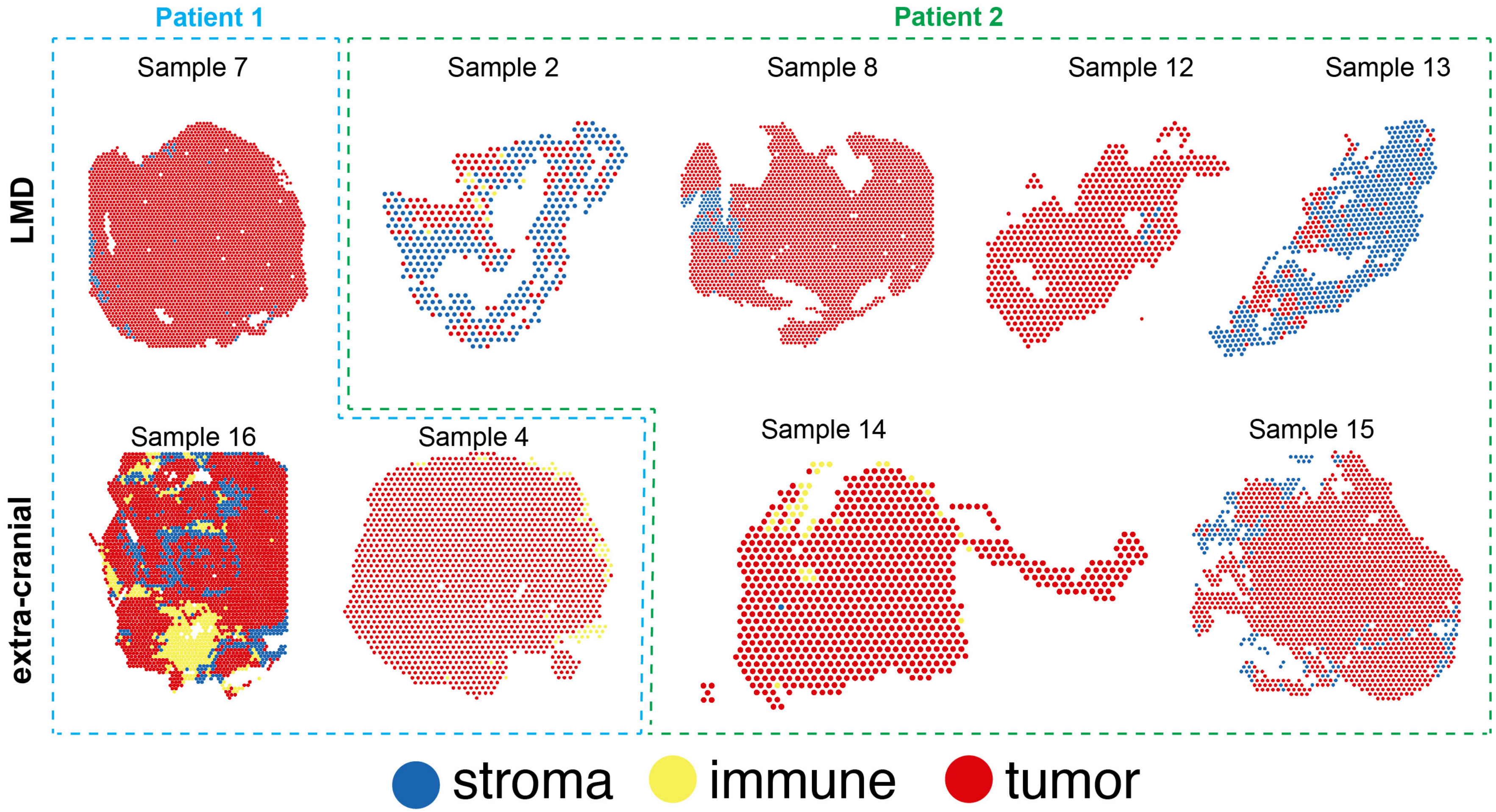

Supplemental Figure 4. Related to Figure 2. Spatial tissue maps showing the position of the stroma, immune, and tumor spots. To better visualize regions of the tumor, stroma and immune infiltration on the spatial maps for consecutive visualizations, the cell subtype categories were condensed into the “tumor”, “stroma” and “immune” categories. Each spot was assigned the stroma, immune or tumor category based on the predominant cell type present.

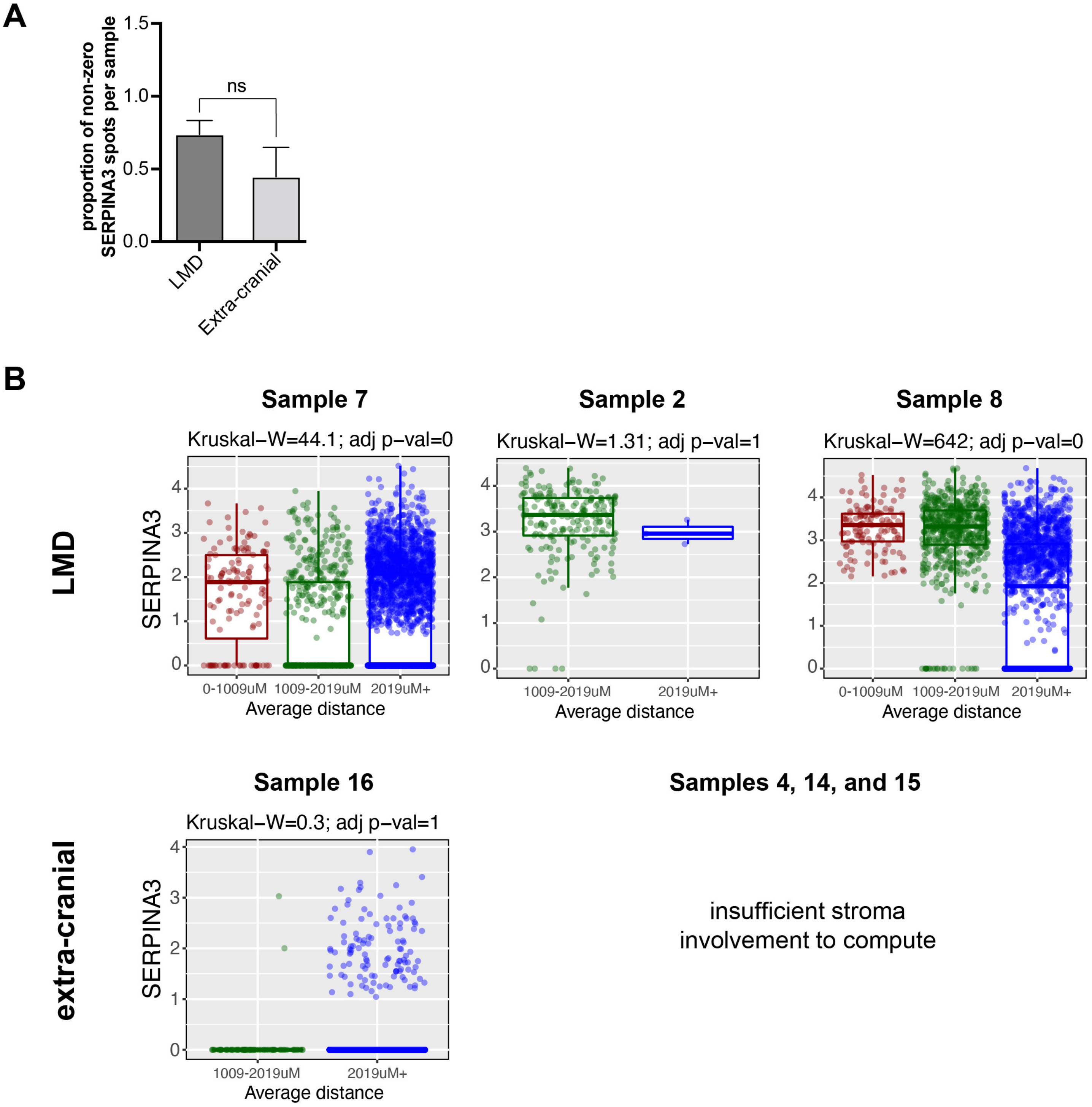

Supplemental Figure 5. Related to Figure 2. **A.** Bar graph showing the proportion of non-zero expression of SERPINA3 spots per sample. Data representative of four biological replicates for extra-cranial disease and five biological replicates for LMD. **B.** Boxplots showing the normalized expression of SERPINA3 gene in each tumor spot based on the average distance between the tumor spot and stroma spots. No enough stromal involvement was found in extra-cranial tissue from Samples 4, 14 and 15 and therefore it was not possible to calculate expression of SERPINA3 relative to distance of tumor to stroma.

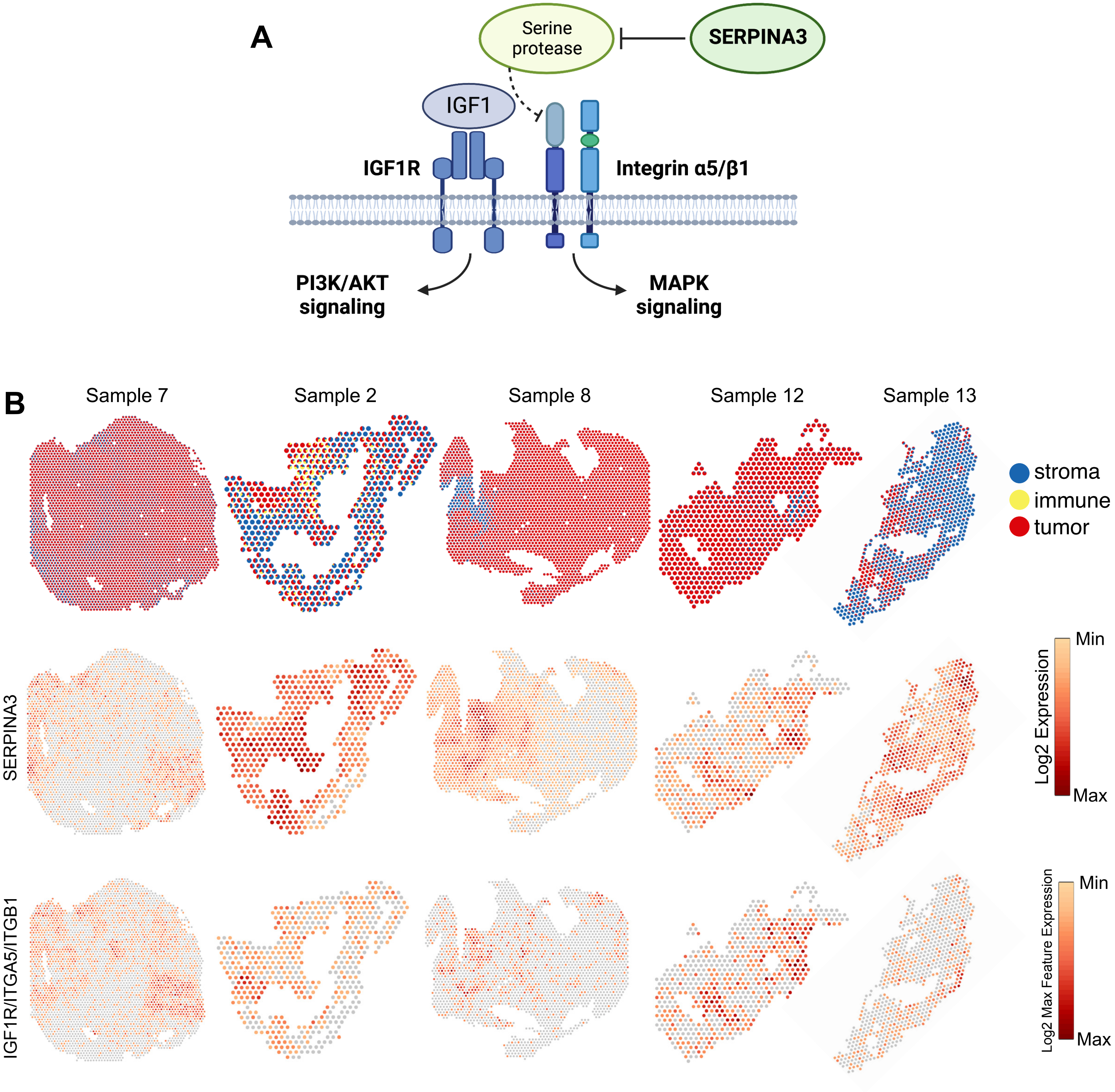

Supplemental Figure 6. Related to Figure 2. **A.** SERPINA3 was previously shown to regulate PI3K and MAPK pathways via activation of the IGF1R/integrinα5β1 signaling[27]. **B.** Spatial tissue maps showing the position of the stroma, immune, and tumor spots along with tissue maps visualizing the log 2 gene expression for SERPINA3, and the log2 max expression for the IGF1R/ITGA5/ITGB1sets of genes.

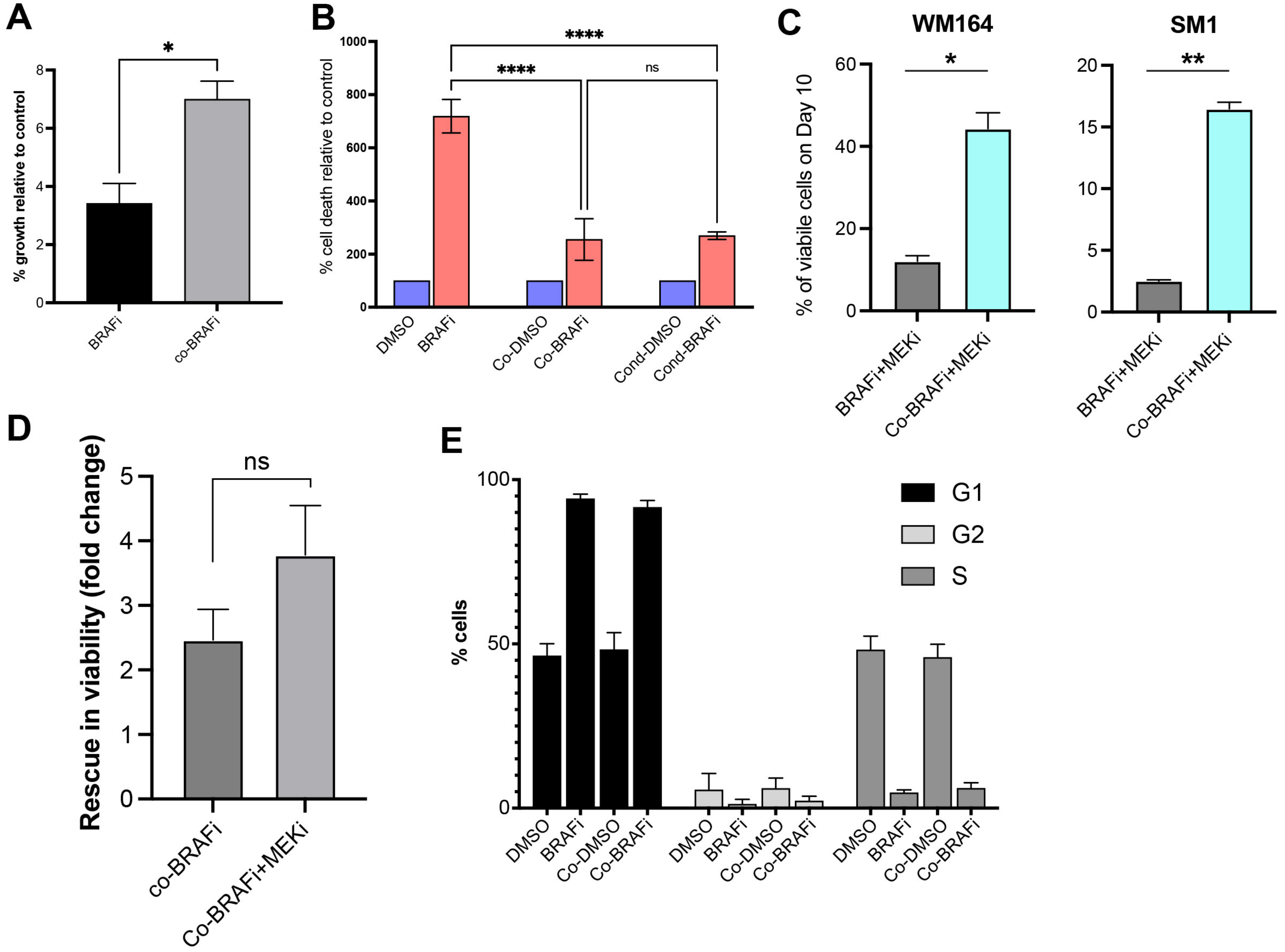

Supplemental Figure 7. Related to Figure 3. **A.** Quantification of % growth following 3μM vemurafenib treatment (BRAFi) in SM1 mono-culture and co-culture with primary murine meningeal cells, relative to day 1 control. **B.** Quantification of viable cells following 3μM vemurafenib treatment (BRAFi) in WM164 mono-culture, co-culture with meningeal cells (Co) and with conditioned media from meningeal cells (Cond). **C.** Bar graphs showing the % of viabile cells left on Day 10 relative to Day 0 for SM1 and WM164 cell lines treated with 100nM dabrafenib + 10nM trametinib. Data representative of two independent biological replicates for BRAFi+MEKi and ten independent biological replicates for BRAFi. **D.** Bar graph showing the magnitude of rescue in WM164 cells from co-culture, displayed as fold change in viability over monoculture conditions treated with 3μM vemurafenib monotherapy versus 100nM dabrafenib + 10nM trametinib combination. Data comparison of Figure 3A and Supplemental Figure 8C. **E.** Flow cytometry assessment of cell cycle using PI staining in WM164 melanoma cells treated with 3μM vemurafenib (BRAFi) or DMSO in the context of monoculture or direct co-culture with primary meningeal cells (Co) under normal media conditions.

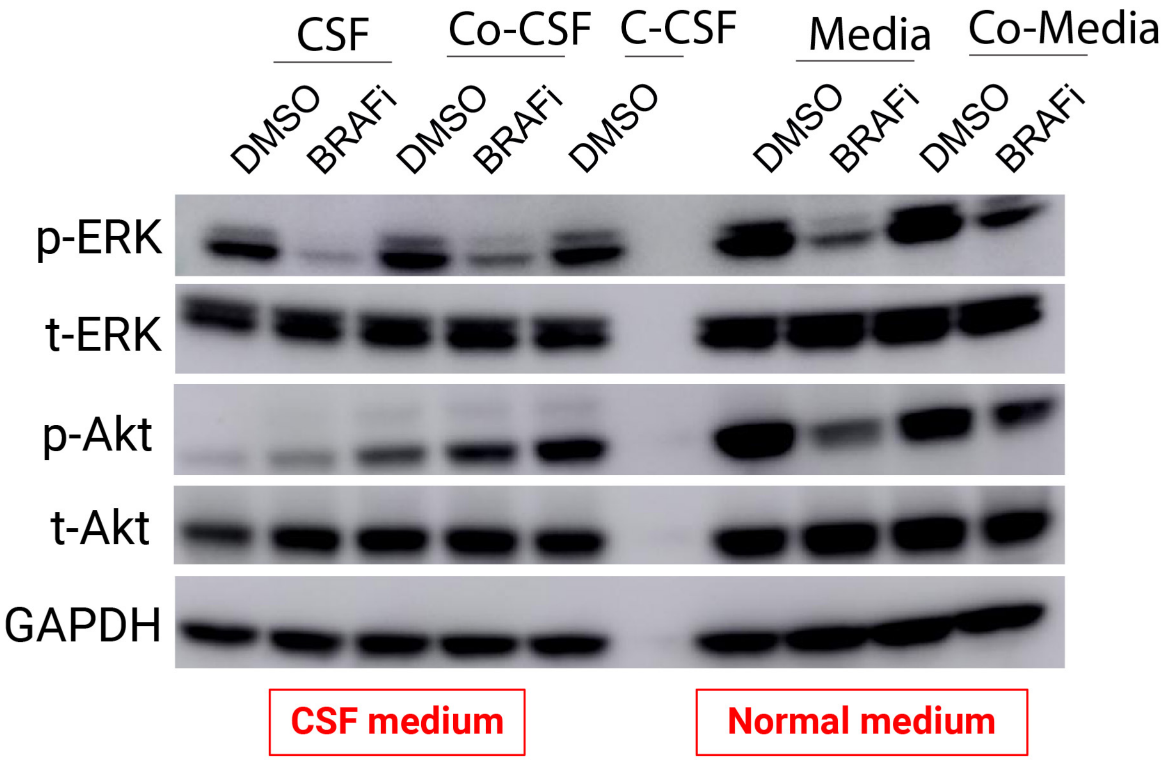

Supplemental Figure 8. Related to Figure 3. Western blot analysis of WM164 melanoma cells treated with 3μM vemurafenib or DMSO control in regular or conditioned media or artificial CSF showing abundance of pERK(Thr202/Tyr204), ERK, pAKT (Ser473) and AKT. Co-CSF is conditioned CSF from co-cultures of melanoma and primary meningeal cells, whereas C-CSF is CSF conditioned by primary meningeal cells only.Co-Media is conditioned media from co-cultures of melanoma and primary meningeal cells.

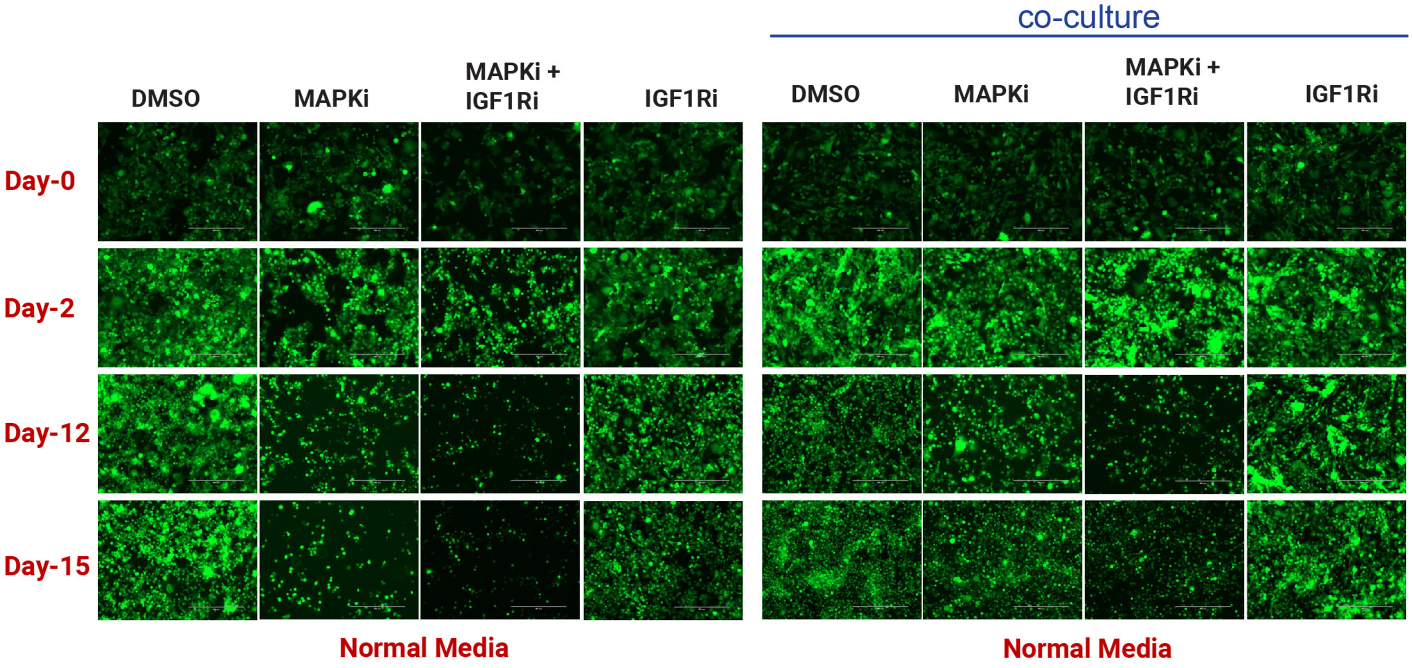

Supplemental Figure 9. Related to Figure 5. Representative microscopy images showing GFP-tagged WM164 melanoma cells treated with DMSO control, 100nM dabrafenib/10nM trametinib (MAPKi), 1µM linsitinib (IFG1Ri), or the triple combination in monoculture versus co-culture with primary meningeal cells in the context of normal media conditions.

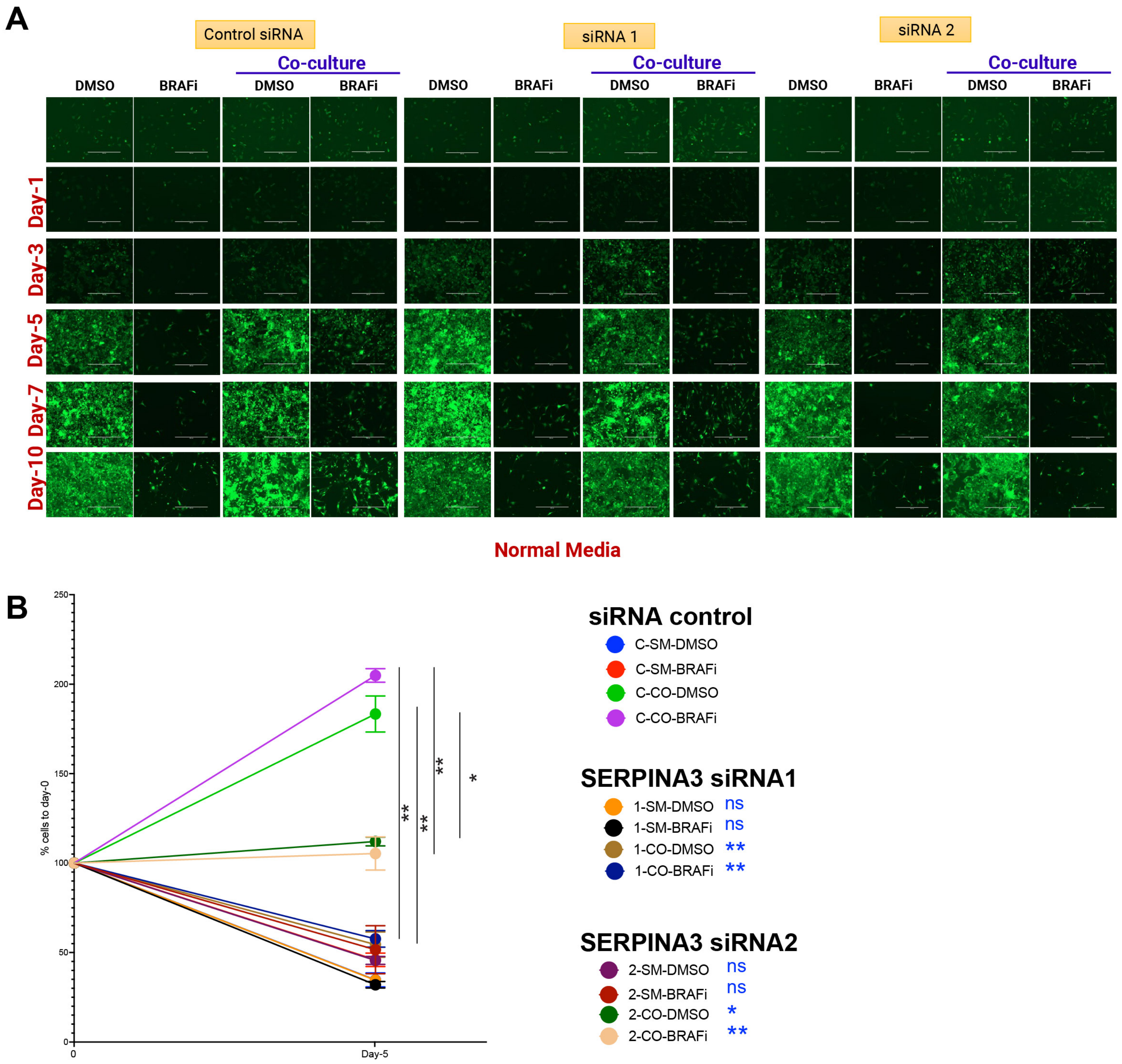

Supplemental Figure 10. Related to Figure 5. **A.** Representative microscopy images showing GFP-tagged WM164 melanoma cells treated with 3µM vemurafenib (BRAFi) or DMSO control in monoculture versus co-culture with primary meningeal cells in the context of normal media conditions following knockdown of SERPINA3 or Control siRNA (siRNAc) in both cell types. **B.** Quantification of data in panel A.

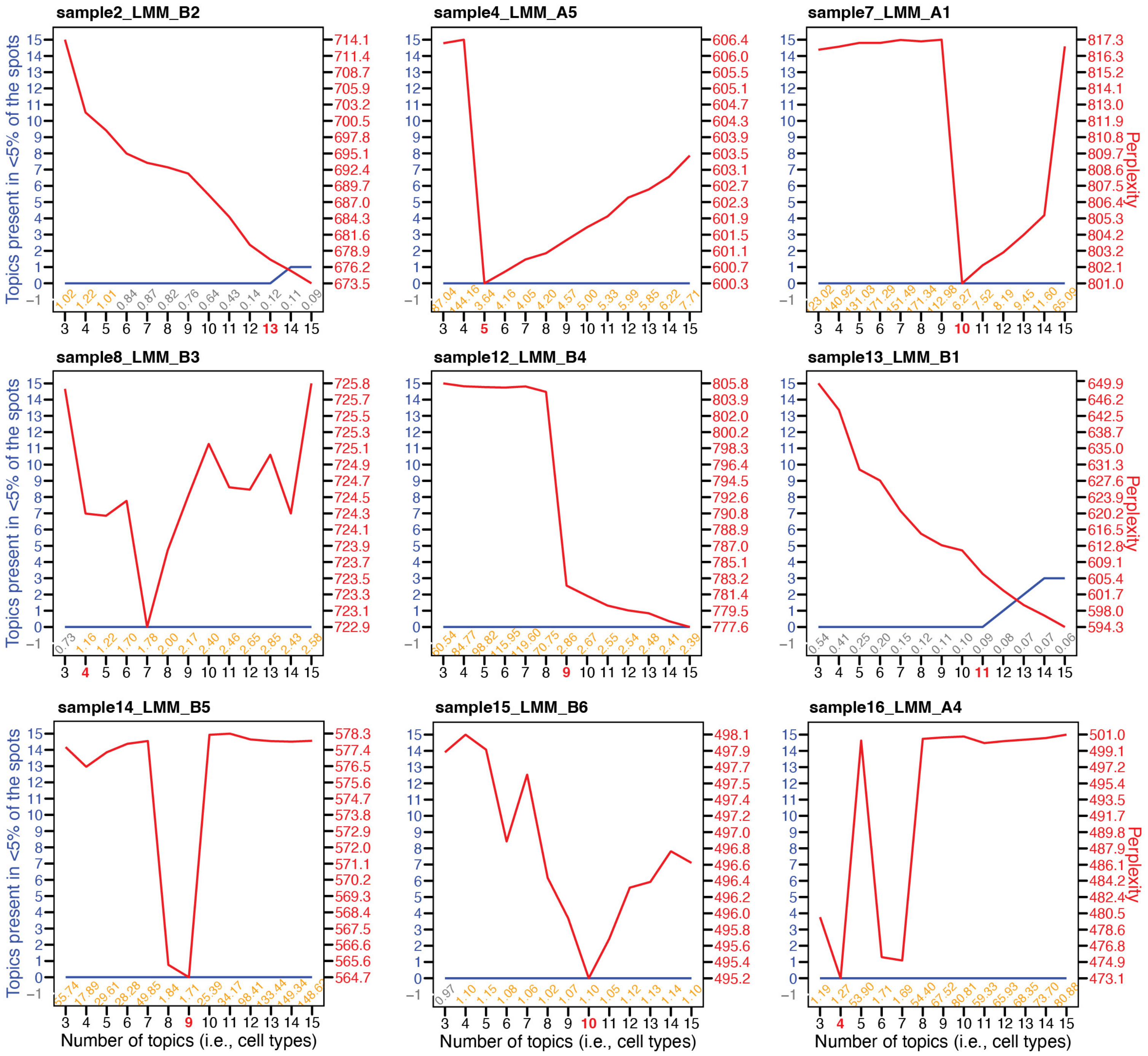

Supplemental Figure 11. Related to STAR Method Identification of cell types and tissue niches (gene expression deconvolution). The perplexity (red line) for a series of LDA models fit to each Visium sample assuming a number of topics ranging from 3 to 15. Models with low perplexity are preferred. To assist our selection of the most likely number of topics, the number of topics scarcely repesented (<5% of spots; blue line) in the model was also examined. A compromise between low perplexity and low number of rare topics was selecte for each sample. The numbers below the curves represent the models' alpha values. Values closer to lower than 1 (gray) are preferred when possible, resulting in each spot having more than one topic. The selected model is shown with a bold red number.

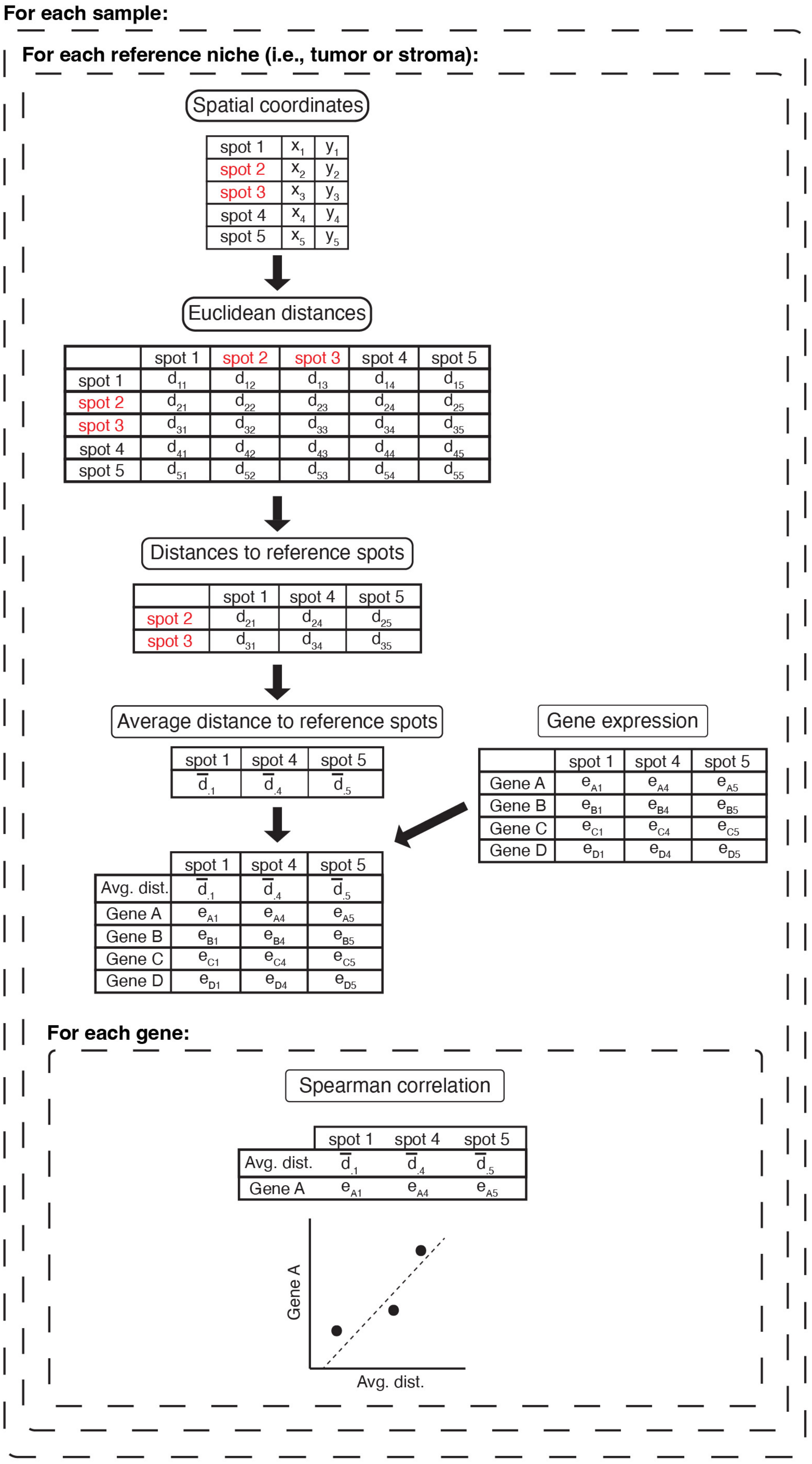

Supplemental Figure 12. Related to STAR Method Gene expression gradients at tissue niche interfaces. The visualization of the algorithm used for calculating gene expression gradients at tissue niche interfaces, described in the methods section.

Supplemental Table 1: Sample cohort characteristics, related to Figure 1.

Sample #	Sample ID	Patient	Source	Site of Disease	Site of sample	Treatment History
Sample 7	LMM_A1	Patient A	Autopsy	LMD	Brain right temporal (LMD)	Targeted therapy
Sample 16	LMM_A4	Patient A	Autopsy	Extra-cranial	4th Hb anterior (R1)	Targeted therapy
Sample 4	LMM_A5	Patient A	Autopsy	Extra-cranial	Sternum	Targeted therapy
Sample 13	LMM_B1	Patient B	Autopsy	LMD	Spinal cord mid (LMD)	Checkpoint inhibition and targeted therapy
Sample 2	LMM_B2	Patient B	Autopsy	LMD	Spinal cord posterior (LMD)	Checkpoint inhibition and targeted therapy
Sample 8	LMM_B3	Patient B	Autopsy	LMD	Brain left temporal (LMD)	Checkpoint inhibition and targeted therapy
Sample 12	LMM_B4	Patient B	Autopsy	LMD	Brain left parietal (LMD)	Checkpoint inhibition and targeted therapy
Sample 14	LMM_B5	Patient B	Autopsy	Extra-cranial	Cecal nodule	Checkpoint inhibition and targeted therapy
Sample 15	LMM_B6	Patient B	Autopsy	Extra-cranial	Right upper chest wall	Checkpoint inhibition and targeted therapy

Supplemental Table 6: Target gene signatures for MAPK and mTOR, related to Figure 2.

MAPK target genes	mTOR target genes	CAF markers
ELK1	SREBF1	PDGFRA
ATF1	MYC	PDGFRB
ATF2	RPS6KA1	ACTA2
MYC	RPS6KA2	S100A4
TP53		PDPN
PAX6		CD70
STAT1		CD74
MAX		FAP
